# Rational design of a vision fusion system with visible and near-infrared spectral integration for improved environmental perception

**DOI:** 10.1093/nsr/nwaf204

**Published:** 2025-05-21

**Authors:** Sen Zhang, Pingdan Xiao, Qinghui Hong, Lin Tang, Zhengdao Xie, Rui He, Bei Jiang, Xitong Hong, Xinjie Li, Haodi Zhu, Ruohao Hong, Chang Liu, Xingqiang Liu, Yawei Lv, Yang Chai, Lei Liao, Xuming Zou

**Affiliations:** Key Laboratory for Micro/Nano Optoelectronic Devices of Ministry of Education & Hunan Provincial Key Laboratory of Low-Dimensional Structural Physics and Devices, School of Physics and Electronics, Hunan University, Changsha 410082, China; College of Computer Science and Electronic Engineering, Hunan University, Changsha 410082, China; College of Computer Science and Electronic Engineering, Hunan University, Changsha 410082, China; Key Laboratory for Micro/Nano Optoelectronic Devices of Ministry of Education & Hunan Provincial Key Laboratory of Low-Dimensional Structural Physics and Devices, School of Physics and Electronics, Hunan University, Changsha 410082, China; Key Laboratory for Micro/Nano Optoelectronic Devices of Ministry of Education & Hunan Provincial Key Laboratory of Low-Dimensional Structural Physics and Devices, School of Physics and Electronics, Hunan University, Changsha 410082, China; Key Laboratory for Micro/Nano Optoelectronic Devices of Ministry of Education & Hunan Provincial Key Laboratory of Low-Dimensional Structural Physics and Devices, School of Physics and Electronics, Hunan University, Changsha 410082, China; Key Laboratory for Micro/Nano Optoelectronic Devices of Ministry of Education & Hunan Provincial Key Laboratory of Low-Dimensional Structural Physics and Devices, School of Physics and Electronics, Hunan University, Changsha 410082, China; College of Semiconductors (College of Integrated Circuits), Hunan University, Changsha 410082, China; Key Laboratory for Micro/Nano Optoelectronic Devices of Ministry of Education & Hunan Provincial Key Laboratory of Low-Dimensional Structural Physics and Devices, School of Physics and Electronics, Hunan University, Changsha 410082, China; College of Computer Science and Electronic Engineering, Hunan University, Changsha 410082, China; College of Computer Science and Electronic Engineering, Hunan University, Changsha 410082, China; Key Laboratory for Micro/Nano Optoelectronic Devices of Ministry of Education & Hunan Provincial Key Laboratory of Low-Dimensional Structural Physics and Devices, School of Physics and Electronics, Hunan University, Changsha 410082, China; Key Laboratory for Micro/Nano Optoelectronic Devices of Ministry of Education & Hunan Provincial Key Laboratory of Low-Dimensional Structural Physics and Devices, School of Physics and Electronics, Hunan University, Changsha 410082, China; College of Semiconductors (College of Integrated Circuits), Hunan University, Changsha 410082, China; Key Laboratory for Micro/Nano Optoelectronic Devices of Ministry of Education & Hunan Provincial Key Laboratory of Low-Dimensional Structural Physics and Devices, School of Physics and Electronics, Hunan University, Changsha 410082, China; Department of Applied Physics, The Hong Kong Polytechnic University, Hong Kong, China; Key Laboratory for Micro/Nano Optoelectronic Devices of Ministry of Education & Hunan Provincial Key Laboratory of Low-Dimensional Structural Physics and Devices, School of Physics and Electronics, Hunan University, Changsha 410082, China; College of Semiconductors (College of Integrated Circuits), Hunan University, Changsha 410082, China; Key Laboratory for Micro/Nano Optoelectronic Devices of Ministry of Education & Hunan Provincial Key Laboratory of Low-Dimensional Structural Physics and Devices, School of Physics and Electronics, Hunan University, Changsha 410082, China

**Keywords:** two-dimensional semiconductor, two-dimensional perovskite, photodetector, vision sensor

## Abstract

With the rapid advancements in autonomous driving, pure vision-based solutions have garnered significant attention. However, existing vision sensors are limited by their specific spectral operating ranges and the complexity of processing hybrid optical/electrical signals. In this study, we present a fully circuit-emulated vision system that employs a vision fusion solution for autonomous driving, integrating image sensing, fusion, edge extraction, and decision-making functionalities. This system utilizes vision sensors featuring an Al_2_O_3_/two-dimensional Ruddlesden-Popper perovskite (2D PVK) heterostructural dielectric and MoS_2_/black phosphorus (BP)/MoS_2_ heterostructural channel, which exhibits persistent nonvolatility and fully light-tunable positive and negative photoresponses when exposed to 1064 nm and 532 nm light, respectively. Notably, when combined with edge extraction circuit design, our vision system achieves all-day visual perception with a 99.0% recognition accuracy for driving scenario information. The integration of the fully circuit-emulated vision system with the vision fusion solution enables a more comprehensive and accurate representation of the driving environment.

## Teaser

This study developed an advanced vision system for autonomous driving that integrates sensing, fusion, edge extraction, and decision-making through a vision fusion approach, enhancing environmental perception and safety in complex driving scenarios.

## INTRODUCTION

Pure vision-based autonomous driving solutions have gained significant attention for their cost-effectiveness, streamlined architecture, and human-like visual recognition and comprehension capabilities [1]. These solutions enable vehicles to drive safely and efficiently by adopting complex image processing and machine learning algorithms that imitate the visual perception mechanisms of human drivers [2,3], and leveraging large-scale, high-performance parallel computing to extract road data, detect obstacles, and interpret traffic signals from video streams [4–7]. Nevertheless, conventional vision sensors, in their endeavor to replicate human vision [8–12], frequently overlook the biological traits and functional constraints of the human visual system. Although human vision excels in processing image information within the visible light spectrum, its performance deteriorates under extreme lighting conditions, intricate background noise, or specific wavelength ranges [13]. In the context of autonomous driving, vehicles must contend with diverse lighting environments [14], spanning from bright daylight to dimly lit nights, as well as interferences from road reflections, vehicle tail lights, and other light sources emitting various wavelengths, which can undermine the precision and reliability of autonomous driving systems [15].

To overcome these challenges, it is crucial that vision sensors possess positive/negative photo-response capabilities spanning the visible to near-infrared (NIR) spectrum [16–19]. First, the visible light spectrum constitutes the primary source of environmental information for autonomous driving systems, providing rich color and texture details crucial for recognizing road signs, vehicles, pedestrians, and more. However, under conditions of intense illumination or backlighting [20], sensors may suffer from overexposure or underexposure, resulting in the loss of image details and subsequently impacting recognition accuracy [21,22]. In this regard, vision sensors equipped with negative photoresponse capabilities can adjust their photoelectric conversion characteristics [17] to mitigate excessive brightness. Meanwhile, NIR images, which capture the thermal radiation of objects, are resilient to such disturbances, thereby compensating for the shortcomings of visible light images [23–25]. However, incorporating fully light-tunable positive and negative photoresponse capabilities across the visible to NIR spectrum in a single device for vision fusion poses a significant challenge. Currently, this issue is being tackled through approaches that involve the integration of electrical and optical signals for image processing [26–31], where matching either the visible spectrum or NIR spectrum necessitates altering the bias or gate voltage [13,32–34], leading to hardware redundancy, additional power consumption, and increased computational latency [35,36]. To the best of our knowledge, an advanced vision system for autonomous driving utilizing a vision fusion solution across the visible to NIR spectrum remains unexplored.

In this study, we present a fully circuit-emulated vision system tailored for autonomous driving applications. The sensor integrates an Al_2_O_3_/2D PVK heterostructural dielectric with MoS_2_/BP/MoS_2_ heterostructural channel, enabling comprehensive functionalities such as image sensing, fusion, edge extraction, and decision-making. Specifically, by harnessing the wavelength-dependent response characteristics resulting from the localized trap sites at the BP/PO_x_ interface and the adjustable ionic migration barrier within the 2D PVK layer, we have successfully achieved continuous non-volatile positive photoconductance (PPC) under 1064 nm light and negative photoconductance (NPC) under 532 nm light. Notably, combined with the design of a novel edge extraction circuit and algorithm, the constructed vision sensor array successfully accomplishes image processing functions that surpass those of the human retina. Compared to pure vision-based solutions with limited operating spectra, the vision sensor array effectively merges visible and NIR visual information, achieving high recognition accuracy of 99.0% in driving scenario information (e.g. humans, cars, dogs, and cats) and maintaining 97.5% accuracy even under non-ideal illuminance conditions. A complete circuit emulation of the proposed vision system, integrating image sensing, weight storage, computing, and decision-making through a vision fusion solution, is achieved. This breakthrough overcomes the development bottleneck of traditional digital signal conversion and computing tasks, enabling vision systems to better comprehend complex visual information and enhancing safety and stability in advanced assisted driving.

## RESULTS AND DISCUSSION

### The proposed vision sensor for image fusion

**Figure 1.**
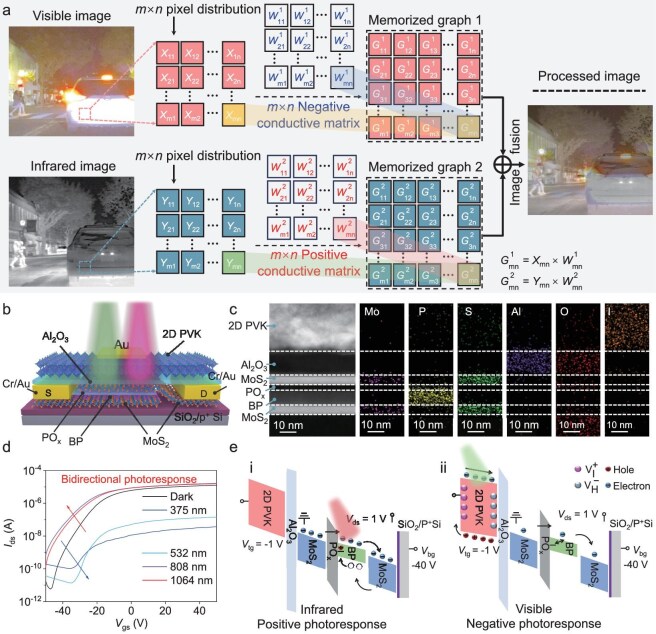
The proposed vision sensor for image fusion and corresponding working mechanism. (a) Illustration of image fusion based on the vision sensor. (b) Device structure of an Al_2_O_3_/2D PVK heterostructural dielectric and MoS_2_/BP/MoS_2_ heterostructural channel. (c) High-resolution transmission electron microscopy and energy dispersive X-ray spectroscopy elemental mapping of the device, which demonstrates clean interfaces. (d) Transfer curves (*I*_ds_–*V*_gs_) of the phototransistor at *V*_ds_ = 1 V under dark conditions and light illumination at different wavelengths with the same light intensity of 100 mW cm^−2^. (e) Energy band diagrams of the device underlying the positive (i) and negative (ii) photoresponse performances.

In complex driving scenarios, pure vision solutions for autonomous driving systems encounter challenges in ineffective target recognition caused by harsh weather conditions, where the vision fusion solutions for stable and reliable assisted driving become increasingly more important [23,37]. To this end, a vision fusion solution based on a vision sensor with bidirectional photoresponse across the visible to NIR spectrum is proposed as shown in Fig. 1a. Specifically, the corresponding target information extraction of NIR and visible images has been used as positive and negative samples, and the stored output results across the two spectrum bands are then calculated to obtain the processed fused image pixel outputs according to the proposed vision fusion strategy. By recombining the m × n pixels (*X*_mn_ and *Y*_mn_, which represent the visible and NIR image brightness distributions, respectively) with the negative conductivity matrix ($W_{{\mathrm{mn}}}^{\mathrm{1}}$) and positive conductivity matrix ($W_{{\mathrm{mn}}}^{\mathrm{2}}$) of the vision sensor and performing image fusion calculations, high-quality and informative fused images are obtained, as shown in Fig. 1a. The road scene images in Fig. 1a are from the RoadScene dataset [38]. This fusion effectively integrates complementary information from both the visible and NIR spectra, enhances image preprocessing capabilities, and substantially accelerates further information processing compared to the original images from each spectrum. Through backend circuit fusion with visible-light signals, this design optimizes energy efficiency without compromising system robustness. Thus, the wavelength-selective photoresponse does not weaken functional completeness but rather enhances task-specific adaptability. To realize this concept, we propose a 2D vision sensor featuring NIR/visible non-volatile PPC and NPC properties. This device is composed of the Al_2_O_3_/2D PVK heterostructural dielectric and MoS_2_/BP/MoS_2_ heterostructural channel (Fig. 1b). The detailed device preparation process is provided in Fig. S1. Here, the upper surface of BP is oxidized through ozone treatment for 4 min. According to density functional theory (DFT) calculations (Fig. S2a–c), the doping of oxygen induces a deep defect energy level in the gap. With decreasing size of the unit-cell along the x-direction, the concentration of O atoms in this direction increases, leading to an increased broadening of the E-k dispersion relation along this dimension, but the broadening is not obvious. Moreover, these defects effectively trap electrons without accelerating their release, owing to weak mutual interactions, indicating their exceptional carrier trapping capability. This, in turn, induces a persistent photoconductivity that exhibits minimal recovery within the experimental timeframes. To further investigate this hypothesis, photoresponse memory behaviors of the devices are characterized based on the ozone treatment time (Fig. S2d). Considering that vacancies, specifically ${\mathrm{V}}_{\mathrm{H}}^{\mathrm{ - }}$, ${\mathrm{V}}_{\mathrm{I}}^{\mathrm{ + }}$, and ${\mathrm{V}}_{{\mathrm{Pb}}}^{{\mathrm{2 - }}}$ (representing hydrogen, iodine, and lead vacancies, respectively), are prevalent defects in organic-inorganic hybrid perovskites and can create acceptor or donor levels [39,40], the Al_2_O_3_/2D PVK heterostructural dielectric is anticipated to effectively promote the capture and prolonged retention of charge carriers. Figure S3 displays the Raman spectra of the MoS_2_/BP/MoS_2_ heterojunction region across all structures, indicating a relatively lattice-damage-free transfer process. Figure 1c shows the high-resolution transmission electron microscopy and corresponding energy-dispersive X-ray spectroscopy elemental mapping images of the device. Here, a seamless and contamination-free heterostructure interface is achieved, which ensures the effective operation of the light-induced field-effect enhancement mechanism within the gate dielectric and facilitates efficient charge transport at the heterointerface. To further evaluate the optoelectrical characteristics of the proposed vision sensor, transfer characteristic curves of the device with and without light illumination at drain-source voltage (*V*_ds_) of 1 V and top-gate voltage (*V*_tg_) of −1 V are investigated (Fig. 1d). Apparently, the device exhibits wavelength-dependent positive/negative photoresponse behavior. Furthermore, we fabricated two reference devices for comparison: one devoid of the Al_2_O_3_/2D PVK heterostructural dielectric, and the other operated with *V*_tg_ of +1 V. Unidirectional photoresponse was observed in these devices, confirming that both the Al_2_O_3_/2D PVK heterostructural dielectric and an appropriate *V*_tg_ are essential for achieving a bidirectional photoresponse, as depicted in Fig. S5a–d.

The fundamental photoresponse behavior of the devices can be elucidated through the operational principle depicted in Fig. 1e. The energy bands of the various materials involved were determined using a combination of Ultraviolet Photoelectron Spectroscopy (UPS), absorption spectroscopy, and DFT calculations (Fig. S4a–i). Specifically, the devices operate as follows: in the absence of light illumination, with bottom gate voltage (*V*_bg_) of −40 V and *V*_tg_ of −1 V applied, the current is restricted by a significant electron-injection barrier induced by PO_x_. Upon 1064 nm illumination (i), the field effect of the Al_2_O_3_/2D PVK heterostructural dielectric on channel current is minimal, owing to poor absorption of NIR light by 2D PVK (Fig. S4a). In this scenario, photoexcited electrons traverse the MoS_2_/BP/MoS_2_ heterostructural channel under applied source-drain bias, while holes remain trapped at the BP/PO_x_ interface due to localized trap sites induced by oxidation. This trapping mechanism provides a photo-gating effect as electrons recirculate within the channel [41,42]. Under 532 nm illumination (ii), vacancy-mediated ionic migration in 2D PVK is accelerated, resulting from a reduction in the activation energy for ionic transport [43]. Photoexcited electrons and anions accumulate at the Al_2_O_3_/2D PVK interface when *V*_tg_ = −1 V, whereas holes and cations migrate towards the gate electrode. This accumulation leads to emergence of an additional negative voltage at the interface, which is responsible for the observed negative photocurrent. When light illumination is terminated, long-lived trapped charges and reduced ionic migration capability contribute to an ultralong positive and negative photocurrent duration, corresponding to non-volatile PPC and NPC, respectively.

### Optoelectronic properties of the vision sensor with non-volatile positive and negative photoconductance

**Figure 2.**
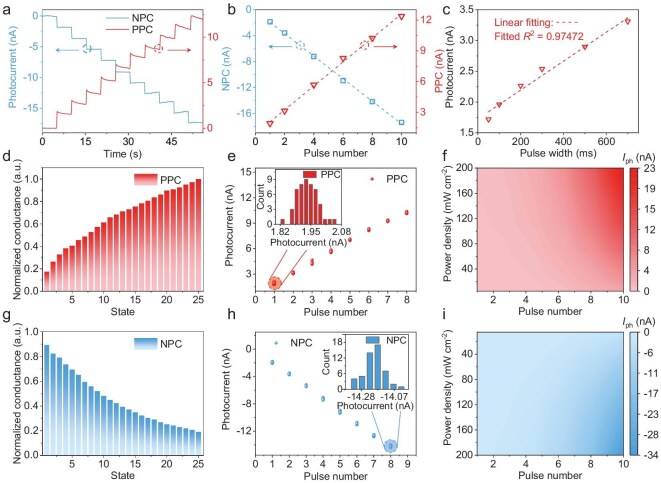
Photoresponse characteristics of the vision sensor. (a) Positive and negative photoconductivity with progressive multilevel states realized by applying periodic 100 ms light pulses both with 5 s intervals. The red curve represents the positive photocurrent and the blue curve represents the negative photocurrent. (b) Extracted cumulative positive/negative photocurrent and the linear fitting. The *R*-squared values of PPC and NPC are 0.99733 and 0.99919, respectively. (c) The modulation of pulse width on the positive photocurrent and the linear fitting. (d, g) Twenty-five discrete conductance states of PPC (d) and NPC (g) triggered by the 1064 and 532 nm light pulse, respectively. (e, h) Conductance distribution at each state for both PPC (e) and NPC (h) stabilization tests when a periodic light pulse is applied. The inset depicts the conductance distribution of a certain state from 50 periods. (f, i) Color mapping of both cumulative PPC (f) and NPC (i) for wavelengths of 1064 and 532 nm, respectively.

To gain a deeper understanding of fundamental photoconductivity properties of the proposed vision sensor, we utilized light sources emitting at wavelengths of 532 nm and 1064 nm to simulate visible light and NIR environments, respectively. As depicted in Fig. 2a, the photocurrent profiles reveal a wavelength-dependent bidirectional photoresponse that emulates the antagonistic shunting and memory functionalities of on/off bipolar cells [12,17]. Notably, both positive and negative photocurrents exhibit non-volatile characteristics, with retention time surpassing 2 × 10^4^ seconds, while also sustaining robust secondary read-out current. This highlights the remarkable fully light-modulated memory capability of the vision sensor (Fig. S6a, b), offering considerable potential for applications in sensing, memory, and advanced cognitive computing [44,45]. As depicted in Fig. 2b, we explored the impact of light pulse number on the non-volatile photocurrent, with dashed lines representing fits yielding *R*² values as high as 0.99733 and 0.99919 for PPC and NPC, respectively. These results affirm the linearity of the photocurrent response to continuous light pulses. Furthermore, comprehensive testing was conducted to assess the response time to light stimuli durations ranging from 50 to 700 ms (Fig. S7a–f). Figure 2c showcases the modulation of photocurrent amplitude by light pulse width, demonstrating a linear relationship akin to that observed with light pulse number (linearity: *R*² = 0.97472). The conductance can be precisely tuned up or down through application of light stimuli of differing wavelengths (Fig. 2d, g). Both PPC and NPC can be programmed into 25 stable and discrete states, offering extensive operational flexibility for tasks such as image fusion, feature extraction, and image recognition using convolutional neural networks (CNNs) [32]. We also investigated the cyclic endurance of PPC and NPC across two tunable operational states. By applying continuous periodic light stimuli for 50 cycles (Fig. 2e, h), we observed that the conductance at a given state remained consistent, with no significant outliers and nearly complete separation between states, highlighting the excellent cyclic stability of the device (see inset and Fig. S8a–f). To ensure reliable device operation across various light intensities, we measured pulse number- and light intensity-dependent photocurrents under 1064 nm and 532 nm light stimuli. Figure 2f, i presents the 2D color maps of PPC and NPC at different wavelengths, revealing a consistent increase in absolute values of the photocurrent with increasing pulse number and light intensity. Additionally, we conducted endurance tests by alternately programming conductance states with light stimuli (Fig. S9a, b). The on and off states were achieved using 1064 nm and 532 nm light stimuli, respectively, and no degradation was observed after 100 cycles of continuous writing/erasing operations, attesting to the reliability for optoelectronic memory applications. Collectively, these findings underscore the considerable potential of the proposed vision sensor for implementing visual sensing, memory, and computation in neuromorphic visual systems.

### Edge extracting computing circuits for feature extraction of fused images

**Figure 3.**
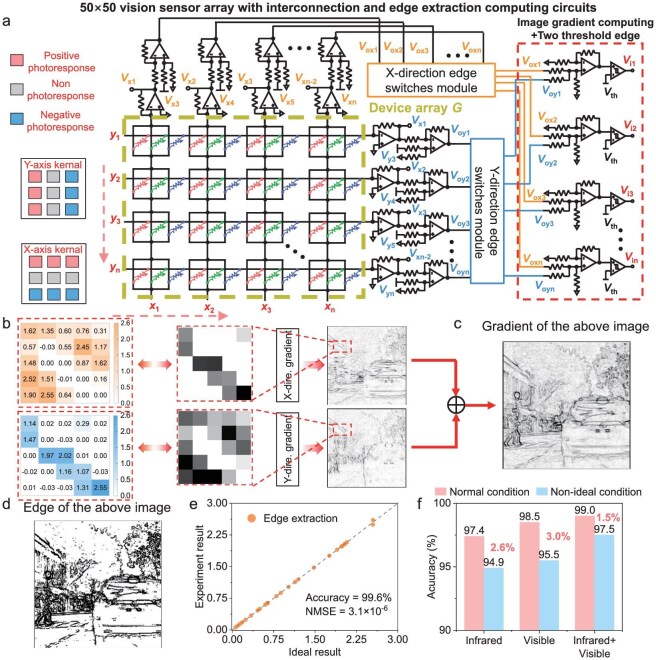
Edge extracting computing circuits for image edge feature extraction based on the vision sensor. (a) Schematic of the proposed edge extracting computing circuits based on the vision sensor array. (b) The X-direction, Y-direction gradients and the pixel block of the example image. (c) The gradient computing result of the example figure by the proposed circuits in the edge process. (d) The final result of the example image calculated by the proposed circuits. (e) The accuracy and NMSE of the proposed edge extracting computing circuits. (f) Comparison between the proposed method and other schemes in the normal and non-ideal condition.

Taking into account the wavelength-dependent photoresponse characteristics, a crossbar configuration is used to form an in-sensor array tailored for image perception, feature extraction, and edge information processing. Each light-sensing unit comprises three devices interconnected in parallel, as depicted in Fig. 3a. The proposed device exhibits distinct current responses across the spectrum from NIR to ultraviolet light, as illustrated in Fig. 1d. This device array, capable of fusing visible and NIR images, functions effectively in both bright and dark conditions. The corresponding modulation mechanism is linear weighted, and we utilize the proposed device to construct a sensing unit for achieving linear weighting in image fusion (Fig. S10). In contrast to arrays composed of single devices, the interconnection strategy of three devices enables trichromatic perception of the visible spectrum. Furthermore, the design of three devices in parallel connection to form a single pixel unit allows for achievement of grayscale color images at the circuit level. This is accomplished by adjusting the operational amplifier (OA) and feedback resistance; the structure of OA and its performance are shown in Fig. S11. Meanwhile, we performed the experiment on the proposed device in the form of 3 × 3 array, which is shown in Fig. S12a–g and is used for demonstrating information fusion between invisible and infrared light. Figure S12c–g shows the example image for the letter ‘H’ of the proposed array. It can be found that the image fusion result by the proposed device preserves the visible characteristics of the optical image while integrating pixel regions that are only observable under infrared light but invisible in the visible spectrum. The device is not limited to its crossbar configuration for image perception, storage, and computation. It can also be arranged in smaller arrays and configured as a receptive field module to perform convolution operations. This versatility stems from positive and negative photoresponses under different wavelengths of light, which align perfectly with practical convolution kernels, with each device mapping to a different parameter.

**Figure 4.**
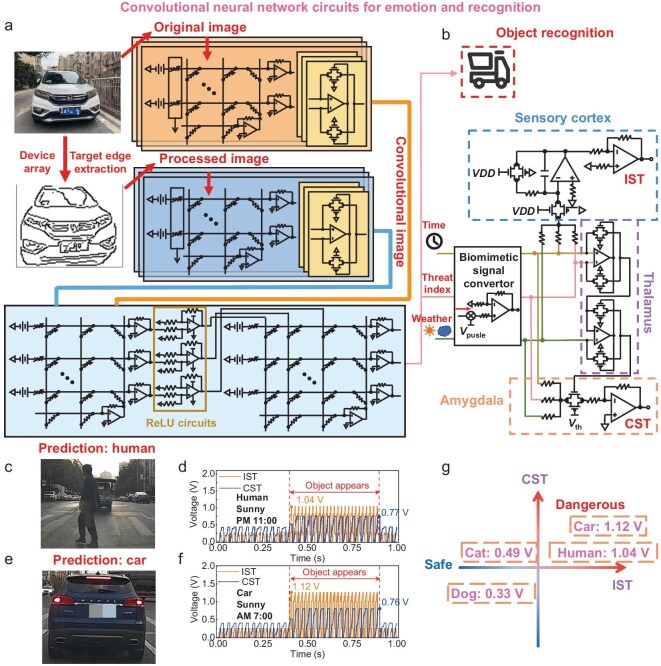
Full circuit emulation implementation of the machine vision system for image recognition and decision-making. (a) Edge extraction and convolution operation for the example image and the proposed CNN circuits. (b) Object recognition result of the example by the proposed circuit and the proposed brain-like circuits based on the emotional dual-loop mechanism. (c, e) Results of object recognition by the proposed CNN circuits. (d, f) Results of the proposed brain-like circuits. (g) Results of the decision made by the proposed brain-like circuits.

Figure 3a illustrates the procedure for executing edge gradient computation utilizing the proposed edge information extraction circuits. According to the principles of image edge extraction, image gradient results are depicted in the form of voltage, encompassing x-orientation and y-orientation gradient outcomes, the detailed principle of which is shown in Fig. S13. Figure 3b exhibits detailed gradient results for x-orientation and y-orientation of the image, as computed based on Fig. 3a. Next, the obtained voltage results *V*_oxi_ and *V*_oyi_ are respectively transferred into an X-direction/Y-direction Edge Switches Module, which is used for managing voltage transmission and wire connection. When the circuit performs an edge extraction task, the corresponding computing process is on the whole parallel, which can achieve reduction in terms of energy and latency due to parallel computing and no-outside-signal-conversion of analog circuit computing. Furthermore, the circuit module in right-hand part of Fig. 3a can achieve dual-threshold edge connection, non-maximum suppression along the gradient direction, and binary image output. First it achieves an entire calculation process of dual threshold edge connection and non-maximum suppression along the gradient direction, and computing the result as shown in Fig. 3c. The corresponding result flows into a comparator, achieving final object edge extraction of the example image Fig. 1a, as shown in Fig. 3d. Therefore, the above circuit is extended to the calculation of the entire image, which has the following principles:


(1) \begin{eqnarray*}
{V}_{{\mathrm{im(i,j)}}} &=& \mathop \sum \limits_{{\mathrm{m = 0}}}^{\mathrm{2}} \mathop \sum \limits_{{\mathrm{n = 0}}}^{\mathrm{2}} {V}_{{\mathrm{EDx(m,n)}}}\,{\mathrm{*}}\,{G}_{{\mathrm{(i - m,j - n)}}}\nonumber\\
&&+\,\mathop \sum \limits_{{\mathrm{n = 0}}}^{\mathrm{2}} \mathop \sum \limits_{{\mathrm{m = 0}}}^{\mathrm{2}} {V}_{{\mathrm{EDy(n,m)}}}\,{\mathrm{*}}\,{G}_{{\mathrm{(i - n,j - m)}}},
\end{eqnarray*}

(2) \begin{eqnarray*}
{{{V}}}_{{\mathrm{i}}\left( {{\mathrm{i,j}}} \right)}{\mathrm{ = }}\left\{ {\begin{array}{@{}*{1}{c}@{}} {{\mathrm{1,}}\quad{{{V}}}_{{\mathrm{im}}\left( {{\mathrm{i,j}}} \right)} \ge {{{V}}}_{{\mathrm{th}}}}\\ {{\mathrm{0,}}\quad{{{V}}}_{{\mathrm{im}}\left( {{\mathrm{i,j}}} \right)}{\mathrm{ < }}{{{V}}}_{{\mathrm{th}}}}, \end{array}} \right.
\end{eqnarray*}

where *V*_EDx(m, n)_ and *V*_EDy(m, n)_ is the voltage representing the Sobel operator, *G*_(i−m, j−n)_ is the conductance of the device array for the captured image, and *V*_th_ is threshold voltage used for optimizing the extracted image. Figure 3e demonstrates that the proposed edge extraction computing circuits exhibit >99.0% computing accuracy, with a normalized mean squared error (NMSE) of 3.1 × 10^−6^. Additionally, the performance of these circuits under various non-ideal conditions was assessed (Figs S14a–c and S15a, b), indicating that the proposed circuits demonstrate good robustness. Building upon these circuits and descriptions, we introduce an in-sensor visual system inspired by the mechanisms of the human brain, designed for security surveillance within driver assistance systems, as depicted in Fig. 4. Our proposed system incorporates a vision and NIR fusion sensor scheme to facilitate auxiliary safe driving. In contrast to vision-only and NIR sensing schemes that rely on vision sensor arrays, our proposed scheme and system attain the highest recognition accuracy under ideal conditions, as illustrated in Fig. 3f. Even when subjected to non-ideal factors such as adverse weather conditions, our system demonstrates minimal accuracy loss compared to other schemes and systems.

### Implementation of machine vision through complete circuit emulation

The driver assistance system is designed based on the devised device and specified requirements. Inspired by the human brain's vision, information processing, and decision-making mechanisms, the system leverages the device to accomplish edge detection and convolution operations, paving the way for subsequent object detection and biologically inspired decision-making. The example image undergoes edge extraction and initial processing through aforementioned device array (Fig. 4a). The resulting processed image is then relayed to the specifically designed CNN circuits for further extraction and enhancement of image features. This employed CNN algorithm has two different functions: the one's output will be converted by peripheral circuits to an electroencephalogram signal and processed by bio-inspired circuits to decide, the other will be executed for object recognition. The CNN training method adopts the Stochastic Gradient Descent method; the detailed process of which is shown in Fig. S16. The parameters of the corresponding convolution kernel are trained off-chip, and weights can be adjusted and mapped onto the device array due to its excellent storage capability (Fig. 2a). The CNN circuits, illustrated in Fig. 4a and Fig. S18, feature a distinct architecture compared to the proposed edge extraction circuits and are composed of a convolution kernel circuit module and full connection layer circuit. The detailed structure of circuits is illustrated in Fig. S18. We also evaluate the proposed CNN circuits under the impact of non-ideal factors, the relative results demonstrate that they are robust (Fig. S20a–c). The proposed brain-like circuits are shown in Fig. 4b, which are inspired by the Dual-Loop Emotional Learning Mechanism. This mechanism simulates the response of an individual when they observe or become aware of something, or encounter a sudden emergency [46]. The corresponding mechanism can be described as follows:


(3) \begin{eqnarray*}
{{{y}}}_{\mathrm{s}}={\mathrm{ max}}\left( {{{{x}}}_{\mathrm{1}}{\mathrm{,\ }}{{{x}}}_{\mathrm{2}}{\mathrm{,\ }}{{{x}}}_{\mathrm{3}}} \right),
\end{eqnarray*}

(4) \begin{eqnarray*}
{{{I}}}_{{\mathrm{danger}}}=\left\{ {\begin{array}{@{}*{1}{c}@{}} {{\mathrm{0,\ }}{{{y}}}_{\mathrm{s}} < {{{v}}}_{{\mathrm{th}}}}\\ {{{A}}{{{x}}}_{\mathrm{1}}+{{B}}{{{x}}}_{\mathrm{2}}+{{C}}{{{x}}}_{\mathrm{3}}{\mathrm{,\ }}{{{y}}}_{\mathrm{s}} \ge {{{v}}}_{{\mathrm{th}}}}, \end{array}} \right.
\end{eqnarray*}

(5) \begin{eqnarray*}
{{{C}}}_{{\mathrm{danger}}}\,{\mathrm{ = }}\,\left\{ {\begin{array}{@{}*{1}{c}@{}} {\displaystyle\frac{{\mathrm{1}}}{{{{RC}}}}\smallint \left( {{{{x}}}_{\mathrm{1}}{\mathrm{ + }}{{{x}}}_{\mathrm{2}}{\mathrm{ + }}{{{x}}}_{\mathrm{3}}} \right){{dt,\ 0 < t}} \le {{{t}}}_{\mathrm{1}}}\\ {{{\mathrm{C}}}_{{\mathrm{danger}}}\left( {{t}} \right){\mathrm{,\ }}{{{t}}}_{\mathrm{1}}{{ < t}} \le {{{t}}}_{\mathrm{2}}}\\ {{\mathrm{0,\ t > }}{{{t}}}_{\mathrm{2}}}, \end{array}} \right.
\end{eqnarray*}

where *x*_1_ represents the biomimetic signal generated by CNN circuits and associated peripheral circuits (Fig. S21); *x*_2_ and *x*_3_, respectively, are time information and weather information, and there is a conversion mechanism for time and weather information (Fig. S22a, b); *I*_danger_ is the instantaneous scenario threat (IST) index generated by brain-like circuits, A, B and C are the setting parameters and *V*_th_ is the setting threshold voltage; *C*_danger_ is the comprehensive scenario threat (CST) index generated by brain-like circuits, *R* and *C* are resistor and capacity of the circuits, respectively, and their multiplication can adjust the speed of the integrator. The detailed working process of the brain-like circuit is depicted in Fig. S21.

The above proposed circuits can provide more precise data for driver assistance systems. Based on the specific requirements of road conditions, these circuits are capable of recognizing four distinct categories: cars, humans, dogs, and cats. The example results are depicted in Fig. 4c, e, presenting a real-world scene captured during driving, showcasing pedestrians crossing the road and vehicle braking ahead, the red annotations indicate the prediction outcomes of CNN circuits, confirming their accuracy in object recognition. The biomimetic signal based on the CNN circuits is illustrated in Fig. S21, which shows the input signal of different objects including humans, dogs, cats, and cars. Figure 4d, f depicts the biomimetic signal of scenarios when humans and cars are present, respectively. It is evident that the output of these circuits remains at low amplitude when humans and cars are not appearing, indicating the absence of any objects or dangerous factors, both instantaneously and over the current period. When humans and cars appear, the objects are captured by the device array and images are processed through edge extraction and CNN circuits. The resulting biomimetic signal is then transmitted to the brain-like circuits. Taking into account time and weather information, which signifies that both IST index and CST index exceed the threshold, prompts the controlled vehicle to take evasive action and sound an alarm. The analysis and results of scenarios are depicted in Fig. S23a–d when a dog and cat appear. Figure 4g presents a two-dimensional chart depicting IST and CST for various object encounters, demonstrating ability of correct inference for object classification. The thresholds for both CST and IST are set at 0.5 V, and the assisted driving system will intervene once these thresholds are exceeded. The figure clearly shows that scenarios involving the sudden appearance of humans or cars exceed the thresholds for both IST and CST. The scenario where the cat suddenly appears exceeds the CST threshold but falls below the IST threshold. Conversely, the scenario where a dog suddenly appears remains below the thresholds for both IST and CST. This is attributed to the similarity between cats and dogs, coupled with the time factor influencing driving safety. The varying CST and IST values prompt the vehicle to adopt different actions in response to changing road conditions. Inspired by the mechanisms of the human brain, the designed brain-like circuits process signals and perform computations in an analog manner, eliminating the need for signal conversion modules and external processors. This design approach achieves a reduction in hardware complexity and an increase in efficiency, integrating visual perception, preprocessing, recognition, and decision-making capabilities in comparison to previous works (see Table S1).

## CONCLUSIONS

We have developed an advanced vision system tailored for autonomous driving, which employs a vision fusion approach integrating Al_2_O_3_/2D PVK heterostructural dielectric and MoS_2_/BP/MoS_2_ heterostructural channel architectures. The vision sensor demonstrates continuous non-volatile PPC and NPC under illumination at 1064 nm and 532 nm wavelengths, respectively, a critical feature that endows it with superior processing capabilities in extreme lighting conditions, surpassing those of the human visual system. Compared to conventional vision-based solutions that are often limited by restricted operational spectra, our system attains a recognition accuracy of up to 99.0% for various driving scenario elements and maintains an impressive accuracy of 97.5% even under challenging lighting conditions. The presented vision fusion strategy enables vision systems to better comprehend complex driving scenarios and make more accurate, holistic decisions in the context of autonomous driving.

## METHODS

### Characterization and measurement of the device

The high-resolution TEM images and element mapping analyses were obtained by a JEOL JEM-2100F TEM/scanning TEM instrument equipped with an Oxford INCA energy-dispersive spectroscopy detector and a Gatan Enfina EELS spectrometer at an operating voltage of 200 kV. Raman spectroscopy measurements were performed using a confocal Raman microscope (Witec alpha300R) under the excitation of a 488 nm laser. The position of the VBM and the work function of the 2D PVK and Al_2_O_3_ films were measured using UPS. The absorption spectrum was also characterized using the UV-Vis spectrometer (Shimadzu UV-2550). The electrical and optoelectrical measurements of the device were characterized by an Agilent B1500A semiconductor parameter analyzer. The incident light sources included lasers with wavelengths of 375, 532, 808 and 1064 nm, respectively. The optoelectronic measurement system incorporates an attenuator for laser power control, a computer-controlled chopper for pulse modulation, a beam expander for uniform illumination, and an aperture to standardize the spot shape/size.

### First-principles calculation

The DFT calculation is performed by an open-source code QUANTUM ESPRESSO. The ultrasoft pseudopotentials are from the pslibrary v1.0.0 and Perdew-Burke-Ernzerhof (PBE) functionals are adopted. The plane-wave cut-off energies for MoS_2_ and BP are 49 and 34 Ry, respectively, according to the library suggestion [47]. An O adsorbed phosphorene system is constructed using a 9 × 4 × 1 phosphorene unit-cell structure, and the decrease in the O atom doping concentration is achieved by increasing the number of P atoms. The k-point sampling at the electronic ground-state calculation is 5 × 5 × 1 and the structures are fully relaxed until the energy variation and atom-forces in the three directions are smaller than 1 × 10^−4^ Ry and 1 × 10^−4^ Ry Bohr^−1^, respectively, at this step. To overcome bandgap underestimation by the PBE functionals, the Heyd-Scuseria-Ernzerhof (HSE) hybrid functional is also adopted [48].

### Algorithm training

The manuscript uses the CNN algorithm to perform object classification and determining dangerous scenarios. We build a picture dataset from a real road scene, which is recorded by a HIKVISION-N6 drive recorder. The CNN algorithm is trained on the NVIDIA GeForce RTX 4060 Ti GPU, and the relative software platform is based on Python 3.9 and Pytorch. The detailed training method and process is illustrated in Figs S16 and S17a–d.

### Validation of full circuit functions

The function validation of the full circuit is emulated by Pspice and Matlab. The circuit design is performed on Pspice, which includes the CNN circuit and brain-like circuit; and the detailed circuit structure is illustrated in Figs 3a, 4a, Figs S18, and S19a, b. We also tested the proposed circuit under some non-ideal factors interference, which is illustrated in Figs S14a–c and S20a–c. The corresponding data computation is based on Matlab and include accuracy and NMSE.

## Supplementary Material

nwaf204_Supplemental_File
