# Supplementary material for: Rational design of a vision fusion system with visible and near-infrared spectral integration for improved environmental perception
Source: Natl Sci Rev. 2025 May 21;12(7):nwaf204. doi: 10.1093/nsr/nwaf204 (PMC12168784; doi:10.1093/nsr/nwaf204)
Supplement: nwaf204_Supplemental_File [file nwaf204_supplemental_file.pdf]

Supporting Information

Rational Design of a Vision Fusion System with Visible and Near-Infrared Spectral Integration for Improved Environmental Perception

Sen Zhang[1,†], Pingdan Xiao[3,†], Qinghui Hong[3,]*, Lin Tang[1], Zhengdao Xie[1], Rui He[1], Bei Jiang[1,2], Xitong Hong[1], Xinjie Li[3], Haodi Zhu[3], Ruohao Hong[1], Chang Liu[1], Xingqiang Liu[2], Yawei Lv[1], Yang Chai[4,]*, Lei Liao[1,2,]* and Xuming Zou[1,]*

[1]Key Laboratory for Micro/Nano Optoelectronic Devices of Ministry of Education & Hunan Provincial Key Laboratory of Low-Dimensional Structural Physics and Devices, School of Physics and Electronics, Hunan University, Changsha 410082, China.

[2]College of Semiconductors (College of Integrated Circuits), Hunan University, Changsha 410082, China;

[3]College of Computer Science and Electronic Engineering, Hunan University, Changsha 410082, China;

[4]Department of Applied Physics, The Hong Kong Polytechnic University, Hong Kong, China

***Corresponding authors**. E-mails: hongqinghui@hnu.edu.cn; ychai@polyu.edu.hk; liaolei@hnu.edu.cn; zouxuming@hnu.edu.cn

[†]Equally contributed to this work.

Keywords: two-dimensional semiconductor, two-dimensional perovskite, photodetector, vision sensor

METHODS

Device Fabrication

The MoS_2 flakes were exfoliated and transferred onto the pre-cleaned P^+-Si substrate with 100 nm SiO_2. The BP flakes were obtained by mechanical exfoliation and transferred onto a SiO_2/P^+-Si substrate. Next, the BP flakes were treated with ozone for 4 min in atmosphere conditions. Subsequently, pre-treated BP flakes containing a PO_x layer were transferred onto MoS_2 by a fixed-point transfer technology. The substrate and the BP/MoS_2 heterostructure were then soaked into the acetone to remove the polymethyl methacrylate film. After that, another MoS_2 flake was transferred onto the BP/MoS_2 sample by performing a similar process. Notably, the top and bottom MoS_2 cannot contact in the designed device structure. Standard electron beam lithography (EBL) was used to pattern the source and drain electrodes, and Cr/Au (10/50 nm) electrodes were deposited by thermal evaporation and standard lift-off process. Then, the atomic layer deposition (ALD) was used to deposit ~12 nm thick Al_2O_3 thin film, and the growth rate of the Al_2O_3 film was 1 Å per cycle. The $(PEA)_2PbI_4$ precursor solution was prepared by dissolving PEAI and PbI_2 in anhydrous N,N-dimethylformamide in a nitrogen-filled glovebox at a 2:1 molar ratio. The mixture was then stirred continuously at 50 ℃ for 10 h. To prepare Al_2O_3/2D PVK heterostructural dielectric, the resulting solution was spin-coated on the Al_2O_3 layer at 4000 rpm for 60 s and the resulting samples were annealed at 100 ℃ for 1 h. Finally, the optoelectronic transistor was realized by thermal evaporation of Au (20 nm) as the top gate electrode.

The fully Circuit Emulation of Machine Vision

The fully circuit design revolves around the proposed devices. Except for the array form based on the proposed device, the circuits design includes the use of peripheral circuits, which facilitate comprehensive system integration and functionality. The circuits are emulation on the Personal Computer, and the emulation platform includes Pspice, Cadence Virtuoso, Matlab and so on. The specifications of the processors running the above software are: RAM size is 15.7 GB, the number of CPUs is 10, and the speed of CPU is 2.50 GHz. The entire emulation process includes programming

mode, verification and performance testing of circuit modules, algorithm training, and validation of fully circuit functions.

Programming mode

The proposed device has two different programming tasks in this work: one is to achieve outside picture perception and storage, another is to map with the weight of trained CNN model. The former depends on the response of the device under different wavelength in Fig. 1d. Considering the existing conductance change curve under different number of light pulses, we can build mathematical model to describe the relation in order to be convenient for the latter requirement based on the Fig. 2d,e. We use drift speed adaptive memristor model to form relation, replace the light pulse with the voltage signal pulse, the relation is as following[1,2]:

$$v(l,t)=\left[\frac{1}{G_{\mathrm{off}}}-x\left(\frac{1}{G_{\mathrm{off}}}-\frac{1}{G_{\mathrm{on}}}\right)\right]\cdot i(t)\#(1)$$

where $v(l,t)$ and $i(t)$ are time-varying voltage and current signal which applied in the device, $v(l,t)$ is effect by the light condition and time. x is the state variable of the device which is normalized broadband conductive region and its value range is [0,1]. G_{on}, G_{off} are respectively the difference between the maximum and minimum conductance and maximum conductance in the device when the state variable $x = 0$. For the derivative of the state variable x, it satisfies the following relationship[1,2]:

$$\frac{\mathrm{d}x}{\mathrm{d}t}=g(i,v)\cdot f(x,i)\#(2)$$

$$g(i,v)=\begin{cases} k_{\mathrm{on}}\left(\frac{1}{G_{\mathrm{off}}}-\frac{1}{G_{\mathrm{on}}}\right)\cdot i(t),\ v(l,t)>v_{\mathrm{on}} \\ 0,\ v_{\mathrm{on}}\leq v(l,t)\leq v_{\mathrm{off}} \\ k_{\mathrm{off}}\left(\frac{1}{G_{\mathrm{off}}}-\frac{1}{G_{\mathrm{on}}}\right)\cdot i(t),\ v(l,t)<v_{\mathrm{off}} \end{cases}\#(3)$$

$$f(x,i)=\begin{cases} [a\cdot(1-x)^{p}],\ \mathrm{sgn}(i)\geq 0 \\ (a\cdot x)^{p},\ \mathrm{sgn}(i)<0 \end{cases}\#(4)$$

where k_{on} and k_{off} are linearly adjustable parameters, which decide that the variation speed of x. The a and p are curve fitting parameters. The v_{off} and v_{on} are the threshold voltages of the device. The entire programming process is program-verify-program. The above operations help us to achieve programming for target conductance.

Based on the above programming mode, matrix-vector multiplication (MVM) under

photo-excitation in the Fig. 3a has the following principle:

$$\Delta I_{\mathrm{n}}=R_{\mathrm{nm}}\times\Delta P_{\mathrm{m}}=\begin{bmatrix} I_1 \\ I_2 \\ \vdots \\ I_{\mathrm{n}} \end{bmatrix}=\begin{bmatrix} R_{11} & R_{12} & \cdots & R_{1\mathrm{m}} \\ R_{12} & R_{22} & \cdots & R_{2\mathrm{m}} \\ \vdots & \vdots & \ddots & \vdots \\ R_{\mathrm{n}1} & R_{\mathrm{n}2} & \cdots & R_{\mathrm{nm}} \end{bmatrix}\begin{bmatrix} \Delta P_1 \\ \Delta P_2 \\ \vdots \\ \Delta P_{\mathrm{m}} \end{bmatrix}\#(5)$$

where I_{n} represents the resultant current the computation outcome, ΔP_{m} denotes the illumination power incident on the n-th device, and R_{mn} signifies the photo-responsivity of the (n,m)-th device.

The minor device array, constituting the convolution kernel module, moves in the crossbar, which represents the image. This module converts the incident light into an electrical signal. With the aid of OA, each MVM result between the convolution kernel and the image, governed by Ohm's and Kirchhoff's laws, yields the convolution output, thereby accomplishing the fundamental operation in edge information extraction.

Verification and performance testing of circuit modules

In the circuit design, the peripheral circuit is mainly designed based on OA. We design a kind of OA based on the TSMC 180 nm node technology, the detailed structure and performance is illustrated in Fig. S11. This work uses the complementary metal-oxide-semiconductor transistors as switches, which is also emulated TSMC 180 nm node technology.

Supplementary Figures:

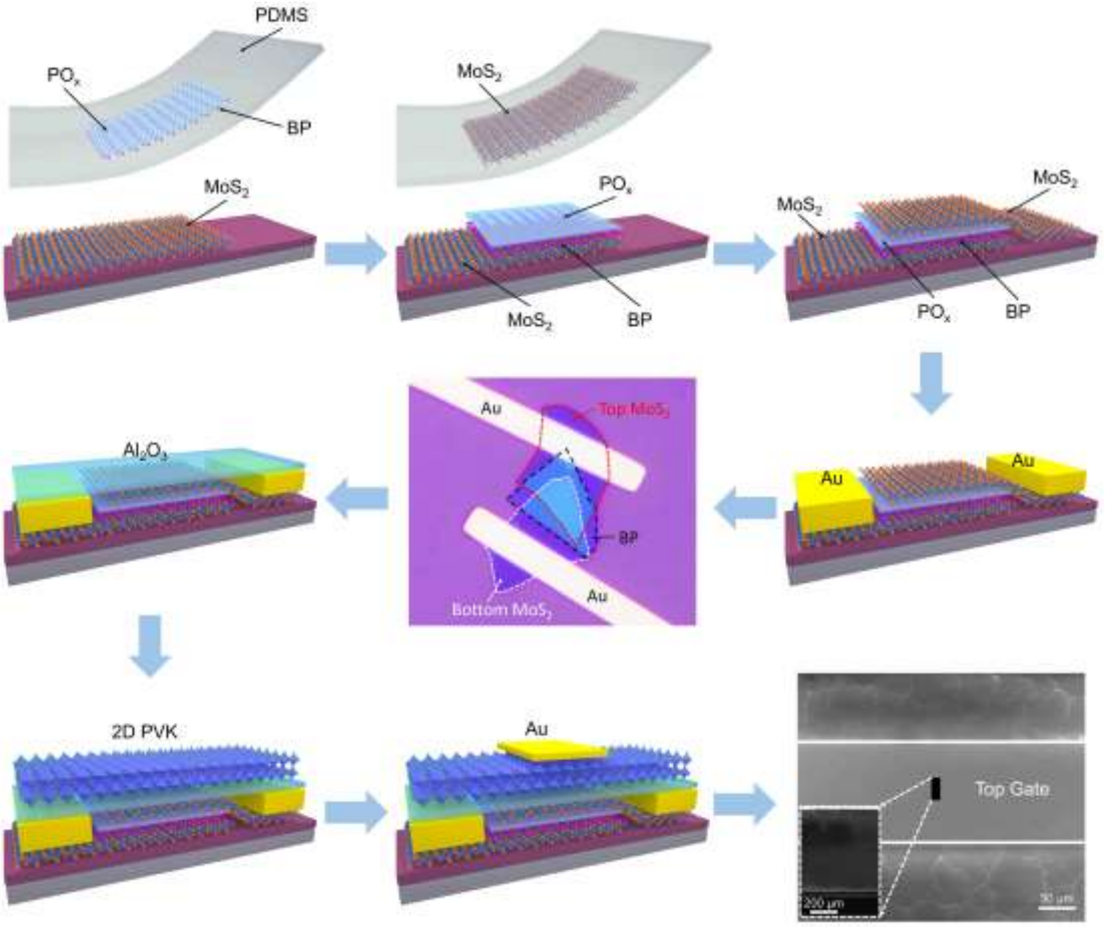

Figure S1. Schematic illustration of fabrication procedure for the MoS_2/BP/MoS_2/Al_2O_3/2D PVK heterostructure device.

Firstly, MoS_2 flakes were mechanically exfoliated from a MoS_2 bulk crystal onto Si substrates with SiO_2 (100 nm thick) as the bottom-gate dielectric. The BP flakes were obtained by mechanical exfoliation from a bulk crystal, and then transferred to a clean SiO_2/P^+-Si substrate. Subsequently, the BP flakes were subjected to oxidation using ozone for 4 minutes to form a superficial PO_x layer. After that, the BP flakes containing the unilateral PO_x layer are again transferred onto MoS_2 by a fixed-point transfer technology. The substrate and the BP/MoS_2 heterostructure were then soaked into the acetone to remove the polymethyl methacrylate film. Another MoS_2 flake was then transferred to the BP/MoS_2 sample by a similar process. We choose 4–5 nm thickness MoS_2 and 12–14 nm BP flakes because the materials with this thickness can

absorb light efficiently while maintaining reasonably high mobility and large signal-to-noise ratio for sensor operation. The drain and source electrodes of Cr (10 nm thick)/Au (50 nm thick) were defined onto the MoS_2/BP/MoS_2 structure by standard electron-beam lithography, followed by thermal evaporation in a vacuum ($<5 \times 10^{-4}$ Pa) and a lift-off process in acetone. Subsequently, a 12-nm thick Al_2O_3 was deposited using ALD. To fabricate the Al_2O_3/2D perovskite heterostructural dielectric, the $(PEA)_2PbI_4$ precursor solution was spin coated on Al_2O_3 layer at 4000 rpm for 60 s and annealed at 100 °C for 1 h. Finally, 20 nm Au electrode as the top gate electrode was deposited on top of the 2D perovskite by metal thermal evaporation with a shadow mask. The top view of the fabricated device is displayed in Fig. S1.

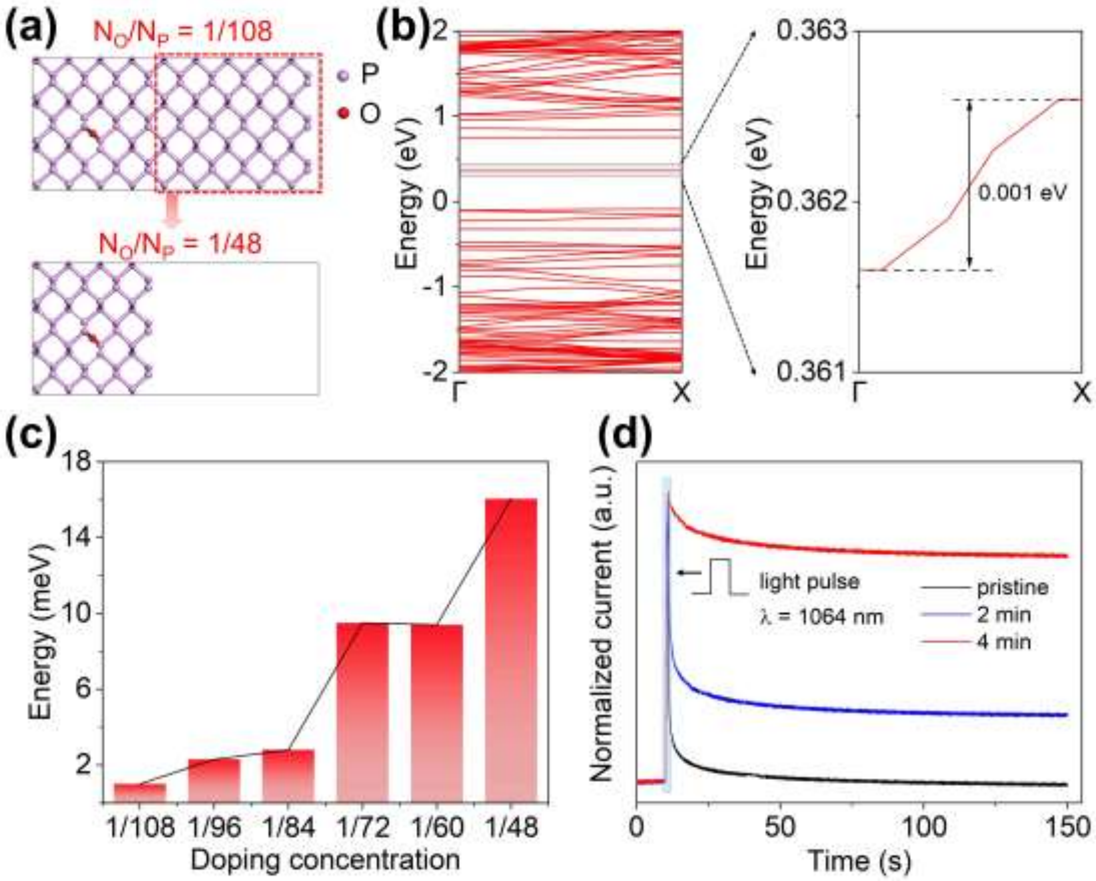

Figure S2. The band structure and photoresponse memory characteristics of oxidized phosphorene. (a) Schematic structure of oxidized phosphorus, wherein the ratio of the number of O atom to the number of P atom is defined as the doping concentration. (b) The calculated band structure of oxidized phosphorene based on DFT calculation. (c) The broadening of the E-k dispersion relation at different doping concentrations. (d) Photoresponse memory characteristics of different ozone treatment times.

We have investigated the energy band structure of oxidized phosphorene with different O atom doping concentrations, where the ratio of the number of O atom to the number of P atom is defined as the doping concentration, and the increase of O atom doping concentration is achieved by decreasing the number of P atoms (Fig. S2a). According to DFT calculations (Fig. S2a,b), the doping of oxygen atom induces deep defect energy level in the gap, where a diagonal bridge with a P-P bond is formed between the most suitable oxygen position and the surface of the phosphorene. The deep defect energy levels located around the oxygen defect and the corresponding E-k dispersion relation are shown in Fig. S2b, c. It is noteworthy that as the size of

unit-cell decreases along the x-direction, the concentration of O atom in that direction increases, leading to an increase in the broadening of the E-k dispersion relation along that dimension, which is not significant. Thus, high density of deep-level defects can be accomplished by oxygen atom adsorption on the surface of phosphorene. Moreover, these defects, upon trapping electrons, do not exhibit accelerated release due to their small mutual interactions, indicating an excellent carrier trapping ability [3,4]. To verify this hypothesis, the photoresponse memory behavior of the devices with different ozone treatment times is further explored, in which the devices with the upper surface of the BP oxidized by ozone treatment for 4 min exhibit significant light-induced memory behavior (Fig. S2d).

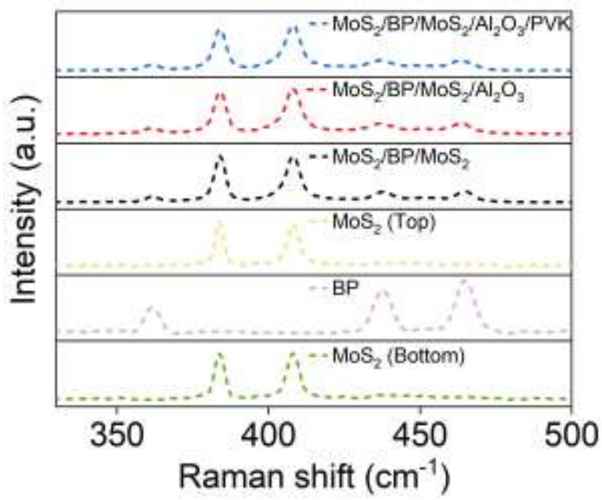

Figure S3. Raman spectra of MoS_2/BP/MoS_2 heterostructure, MoS_2/BP/MoS_2/Al_2O_3, and MoS_2/BP/MoS_2/Al_2O_3/2D PVK structures.

Raman spectra measured in the heterojunction region contain peaks from both BP and MoS_2 (Fig. S3). Meanwhile, the corresponding Raman spectra of the MoS_2/BP/MoS_2 heterojunction regions across all structures are given in Fig. S3, where no obvious variation in the peak positions is observed, indicating a relatively lattice-damage-free transfer process.

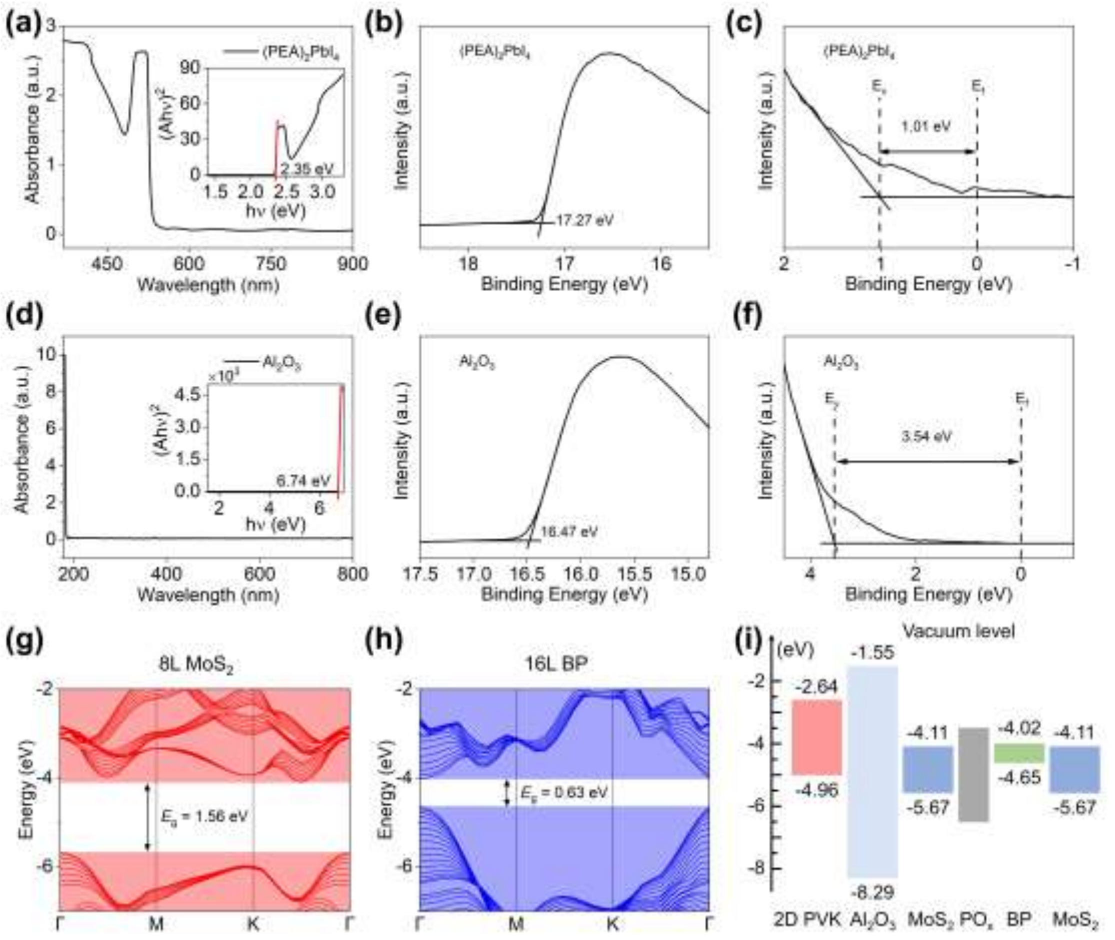

Figure S4. The energy band diagram of the MoS_2, BP, 2D PVK and Al_2O_3 layers. (a) The absorption spectrum and corresponding Tauc plot of the 2D PVK layer for calculating the optical band gap. (b) UPS binding energy profiles of 2D PVK layer, showing the secondary electron cut-off energy associated with the work function. (c) UPS binding energy profiles of 2D PVK layer, showing the valence band region. (d) The absorption spectrum and corresponding Tauc plot of the Al_2O_3 layer for calculating the optical band gap. (e) UPS binding energy profiles of Al_2O_3 layer, showing the secondary electron cut-off energy associated with the work function. (f) UPS binding energy profiles of Al_2O_3 layer, showing the valence band region. (g) The detailed band structure of the used 8 layer MoS_2 (8L MoS_2) obtained from DFT calculation. (h) The detailed band structure of the used 16 layer BP (16L BP) obtained from DFT calculation. (i) The energy band alignment of the device structure before contact.

The optical band gaps and band structures of 2D PVK and Al_2O_3 were determined

using ultraviolet-visible absorption spectroscopy and Ultraviolet Photoelectron Spectroscopy (UPS) with a photon energy of 21.22 eV. The band gaps of the 2D PVK and Al_2O_3 are estimated from the Tauc plots in the inset of Fig. S4a,d (i.e., the curve of converted $(\alpha h\upsilon)^r$ vs $h\upsilon$ from the absorption spectrum, where α is the absorption coefficient, h is the Planck's constant, υ is the light frequency, $r = 2$ for a direct-bandgap material, and $r = 1/2$ for an indirect-bandgap material) [5]. Thus, the band gaps of the 2D PVK and Al_2O_3 can be calculated as 2.32 eV and 6.74 eV, respectively, by measuring the x-axis intercept of an extrapolated line from the linear regime of the curve. The valence band maximum (VBM) and conduction band minimum (CBM) for 2D PVK (Fig. S4b,c) and Al_2O_3 (Fig. S4e,f) can be further calculated from UPS binding energy profiles with VBMs and CBMs of −4.96 eV (−8.29 eV) and −2.64 eV (−1.55 eV), respectively. The detailed energy band structures of the MoS_2 and BP calculated with DFT are shown in Fig. S4g,h, the calculated E_g for 8L MoS_2 and 16L BP are 1.56 and 0.63 eV, respectively. Figure 4i further shows the energy band alignment of various materials before contact.

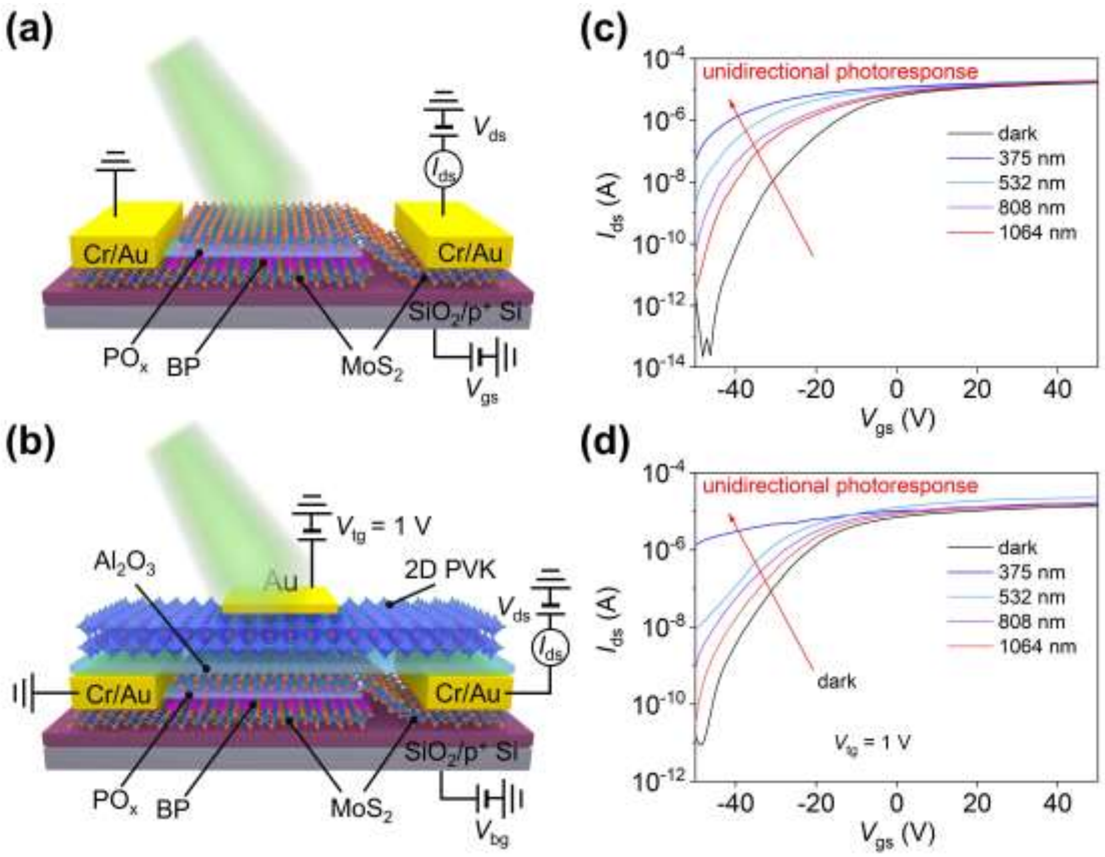

Figure S5. Transfer characteristic curves and structure of two reference devices. (a) Device structure of MoS_2/BP/MoS_2 heterostructure device. (b) Device structure of Al_2O_3/2D PVK heterostructural dielectric and MoS_2/BP/MoS_2 heterostructural channel architectures. (c) Transfer curves of the MoS_2/BP/MoS_2 heterostructure device at V_{ds} = 1 V under the dark condition and light illumination at different wavelengths, showing that the devices exhibit unidirectional photoresponse behavior. (d) Transfer curves of the vision sensor at V_{ds} of 1 V and V_{tg} of 1 V under the dark condition and light illumination at different wavelengths with same light intensity of 100 mW cm^{-2}, indicating that the device exhibits unidirectional photoresponse performance.

To evaluate the optoelectrical characteristics of two reference devices without Al_2O_3/2D PVK heterostructural dielectric or V_{tg} with of +1 V, transfer curves of the devices at V_{ds} = 1 V under the dark condition and light illumination at different wavelengths are investigated, and the unidirectional photoresponse was only observed from the two devices, as shown in Fig. S5. Specifically, the MoS_2/BP/MoS_2 heterostructure device exhibits a unidirectional photoresponse under light illumination

(Fig. S5c), and the corresponding device structure is depicted in Fig. S5a. When V_{bg} of −40 V and V_{ds} of +1 V are applied without light illumination, the current is restricted by the significant electron-injection barrier induced by PO_x. Upon light illumination, the photoexcited electrons transport in MoS_2/BP/MoS_2 heterostructural channel under the applied source-drain bias, while holes remain trapped at BP/PO_x interface. This trapping mechanism provides a photo-gating effect as electrons recirculate within the channel, which is responsible for the observed positive photocurrent. Another reference device with V_{tg} of +1 V exhibits a unidirectional photoresponse under light illumination (Fig. S5d), and the corresponding device structure is depicted in Fig. S5b. Photoexcited holes and cations accumulate at Al_2O_3/2D PVK interface when V_{tg} = +1 V, whereas electrons and anions migrate towards the gate electrode. This accumulation leads to the emergence of an additional positive voltage at the interface, which is responsible for the observed positive photocurrent.

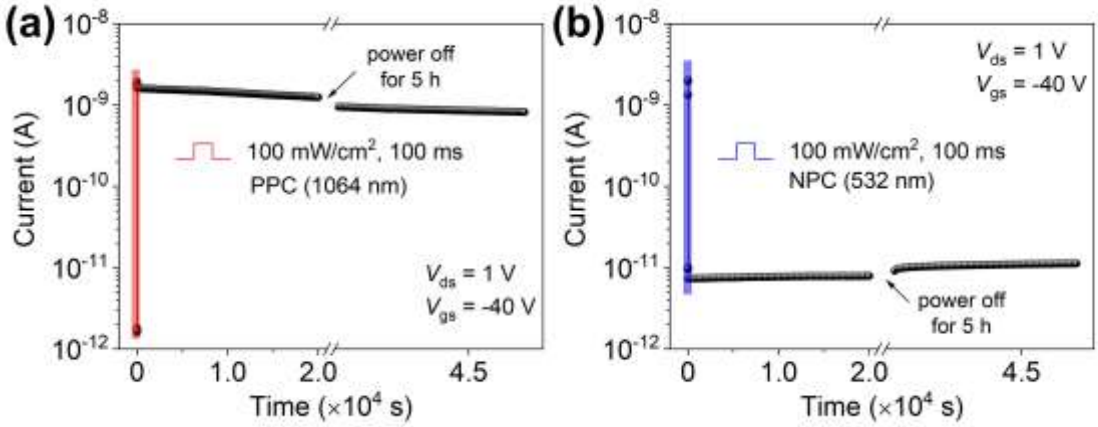

Figure S6. Optoelectronic storage performances of PPC and NPC. (a) The retention of PPC is demonstrated under a 1064 nm laser pulse. The laser durations for PPC are 100 ms. (b) The retention of NPC is demonstrated under a 532 nm laser pulse. The laser durations for PPC are 100 ms.

When the device is illuminated by a 1064 nm laser for 100 ms, the readout current rises rapidly (Fig. S6a). When the laser is turned off, the current can be fully maintained in the measurement range of 2.5×10^4 s. Even after a full power off of 5 h, a robust secondary readout current is maintained. Similarly, under illumination by a 532 nm laser for 100 ms, the readout current decreases rapidly (Fig. S6b). After the laser is turned off, the current continues to exhibit a robust secondary readout current even after 5 hours of being fully powered off.

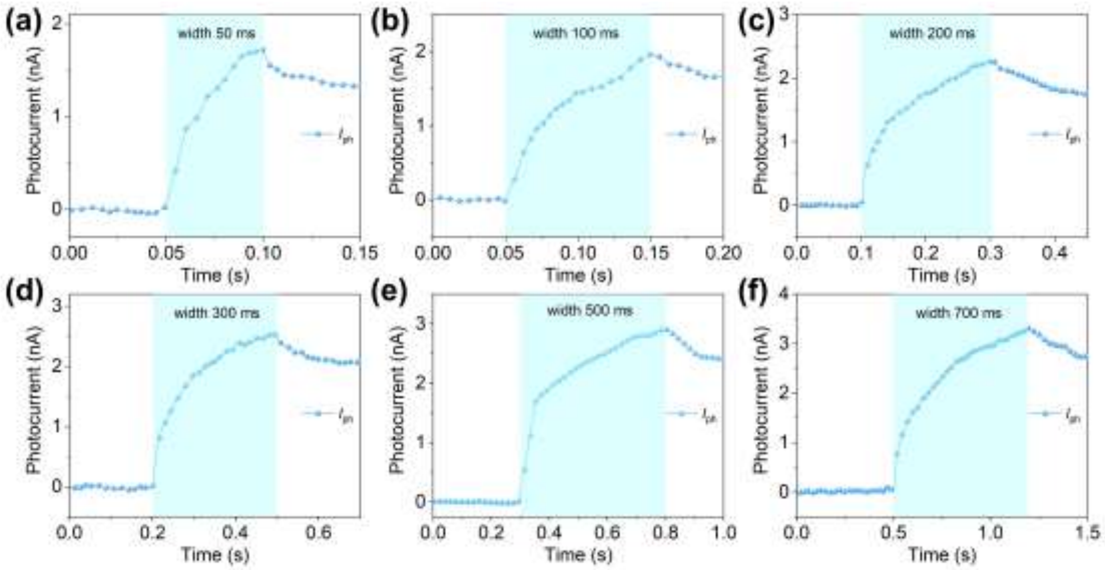

Figure S7. The photocurrent with different duration of stimuli from 50 ms to 700 ms. The durations are selected as (a) 50 ms, (b) 100 ms, (c) 200 ms, (d) 300 ms, (e) 500 ms, (f) 700 ms, respectively.

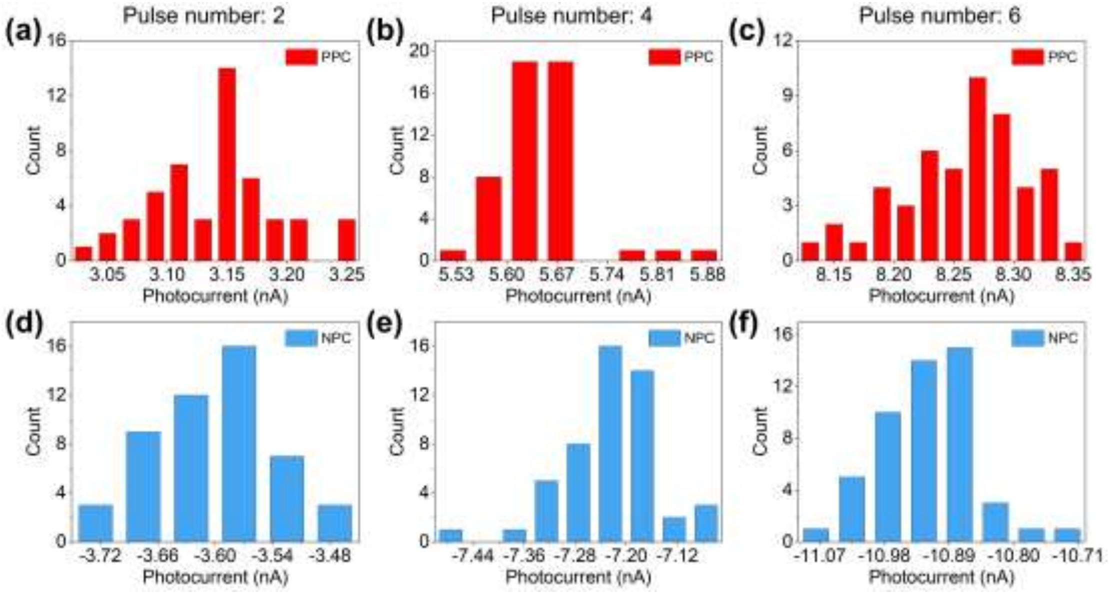

Figure S8. The distribution of different states from 50 periods for both PPC and NPC. (a-c) The pulse number for PPC are selected as (a) 2, (b) 4, (c) 6, respectively. (d-f) The pulse number for NPC are selected as (d) 2, (e) 4, (f) 6, respectively.

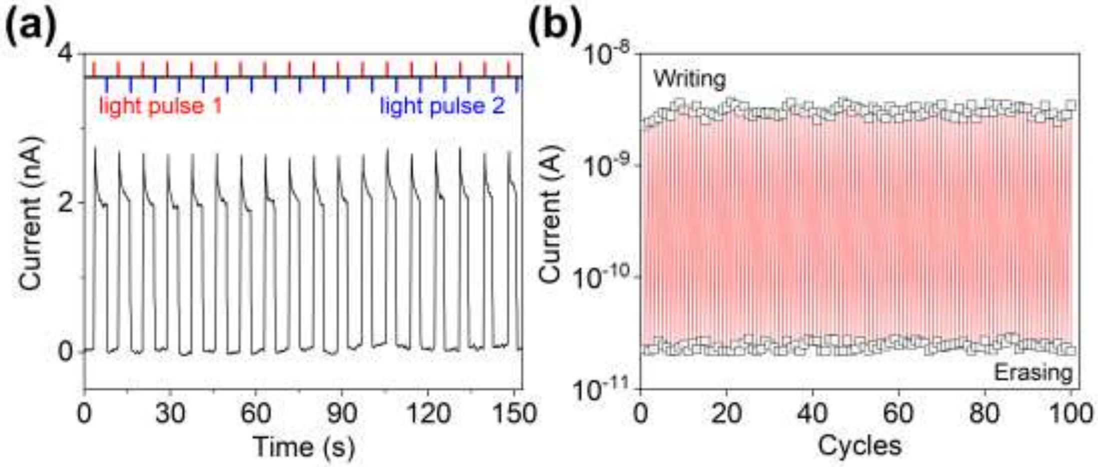

Figure S9. The endurance measurements of the device. (a) The writing, storing, and erasing process of the device with multiple operating cycles. The on and off state current of the device was achieved by applying 1064 nm and 532 nm light stimuli respectively. The number of switching cycles is 18. (b) The endurance test of the device during 100 continuous writing/erasing operation.

Figure S9a depicts the dynamic operation of the device, encompassing optical programming (1), on-state reading (2), optical erasing (3), and off-state reading (4). Upon activation of the 1064 nm laser pulse, the device swiftly transitions to a highly conductive state. This conductive state remains stable for on-state reading after the laser is extinguished. Conversely, when the 532 nm laser pulse is applied, the device promptly reverts to its initial low-conductive state. Following laser cessation, this low-conductive state persists, facilitating off-state reading. Notably, no significant degradation was observed over 100 cycles, demonstrating the robust reliability of the device, as illustrated in Fig. S9b.

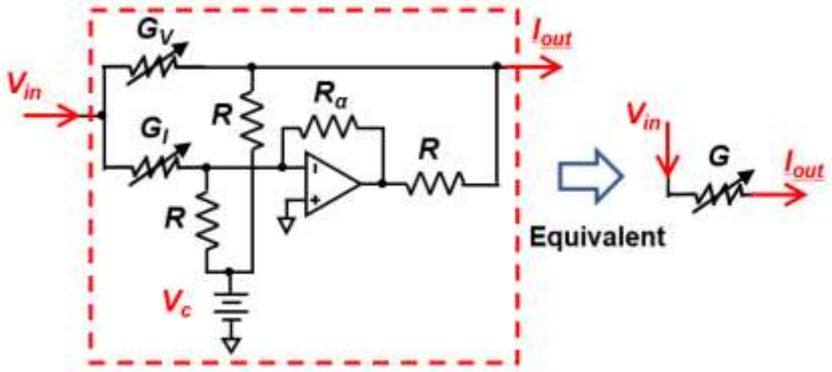

Figure S10. The structure of the sensing cell based on the proposed device, which is used for image fusion.

In the proposed device, it can have the corresponding response for different condition. As shown in Fig. 1d, it can achieve negative photoresponse under visible light and positive photoresponse under infrared light. In there, we propose an image fusion mechanism based on the proposed device, which enables the fused image to not only retain the color information of visible light but also reveal hidden object information that can only be observed under infrared light. The corresponding modulation mechanism is linear weighting, and we utilize the proposed device to construct a sensing unit for achieving linear weighting in image fusion.

As illustrated in Fig. S10, the proposed sensing cell compose of the proposed devices, resistance and OA, and the cell has the following relation:

$$\begin{cases} I_1 = \dfrac{\left(V_{in} G_I - \frac{V_c}{R}\right) R_\alpha}{R} \\ I_{out} = V_{in} G_V - \dfrac{V_c}{R} + I_1 \end{cases} \#(6)$$

where G_V and G_I respectively represent the device sensing visible and infrared light information, $R\alpha$ is the resistance representing the coefficient of the modulation mechanism and V_c is the compensate voltage. The sensing cell has the following modulation mechanism for fusion:

$$G_E = \Delta G_V - \alpha \Delta G_I \#(7)$$

In there, we use V_c and resistance to obtain current to extract the changing information of device, and the OA is used to multiply the infrared information by the

coefficient α. The sensing cell is equivalent to sensing device and is used in the array for assisting image fusion.

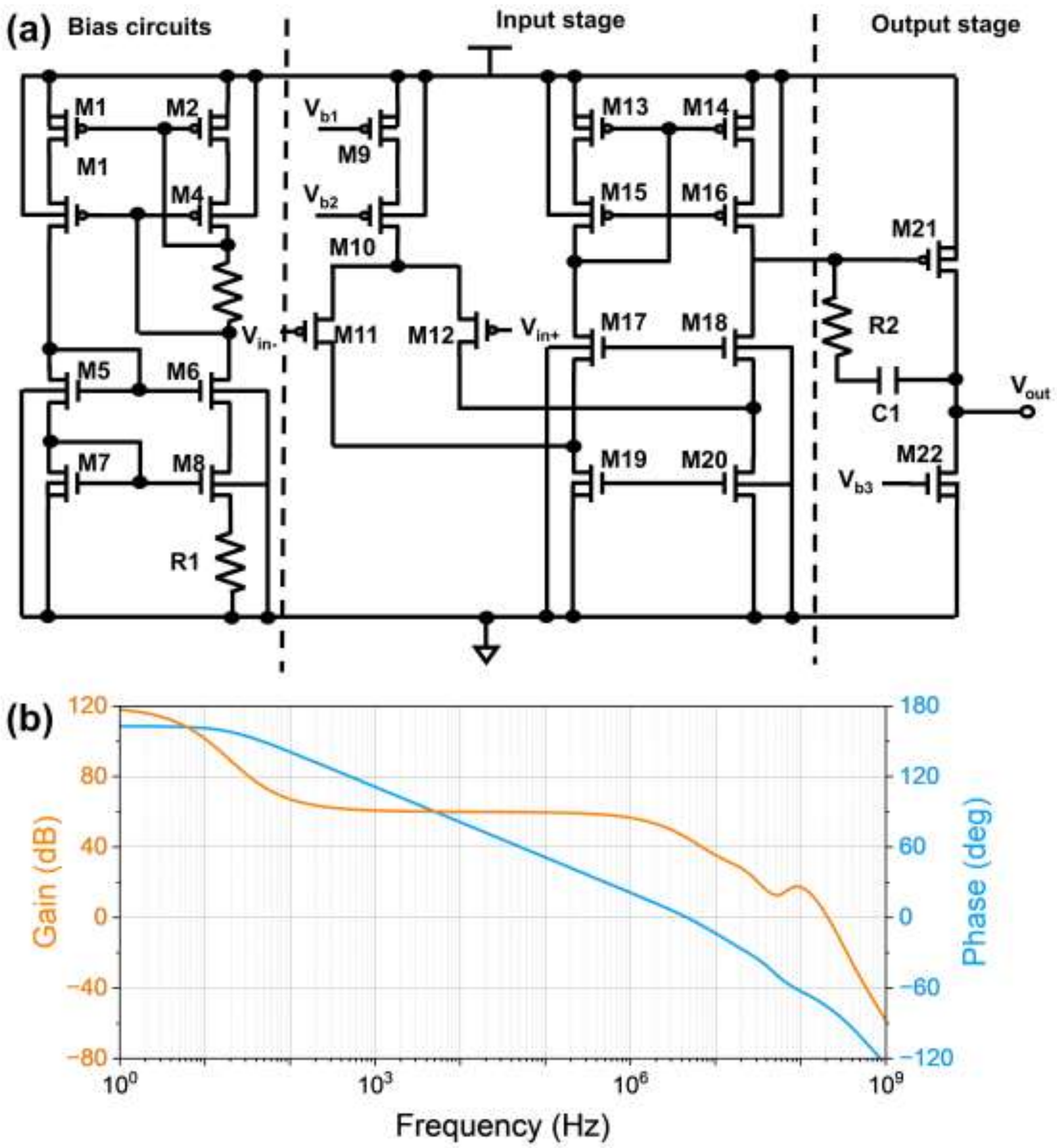

Figure S11. The structure and performance of the utilized operational amplifiers. (a) The structure of the utilized operational amplifiers. (b) The designed operational amplifiers have 108.5 dB open-loop gain.

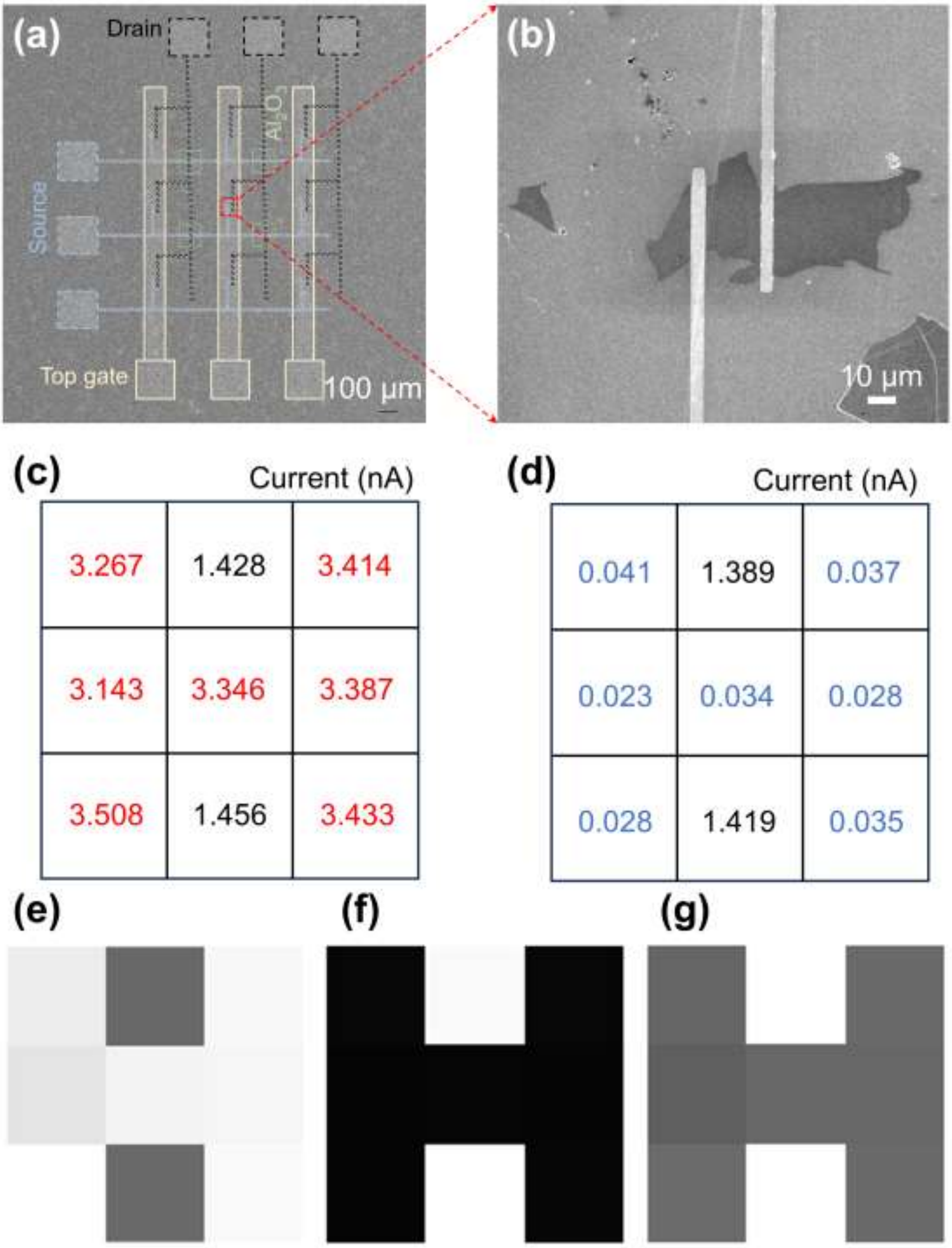

Figure S12. Demonstration of information fusion between visible and infrared spectra with a 3×3 sensor array. (a) SEM image of 3×3 sensor array. (b) SEM image of a single MoS_2/BP/MoS_2 device. (c) Current of the sensor array irradiated by a 1064 nm light pulse. (d) Current of the sensor array irradiated by a 532 nm light pulse. The sensor array was operated at V_{bg} = -40 V, V_{tg} = -1V, V_{ds} = 1 V with a light pulse intensity of 100 mW/cm^2 and a duration of 100 ms. (e) Letter H image under infrared

light. (f) Letter "H" image under visible light. (g) Image fusion of Letter "H" image by the proposed device and mechanism.

We first clarify the fabrication process of the 3×3 sensor array. The process begins with mechanically exfoliated MoS_2 flakes transferred onto a pre-cleaned SiO_2/P^+ -Si substrate (100 nm SiO_2 layer), followed by the transfer of a BP flake onto the same substrate. The BP flake undergoes a 4-minute ozone treatment under ambient conditions to form a controlled PO_x layer, after which it is precisely aligned and transferred onto the MoS_2 layer using a fixed-point transfer technology. Residual polymethyl methacrylate (PMMA) is removed by acetone immersion, and a second MoS_2 flake is transferred onto the BP/MoS_2 heterostructure, ensuring physical isolation between the top and bottom MoS_2 layers to prevent electrical interference. To construct the 3×3 sensor array, adjacent MoS_2/BP/MoS_2 units are spaced ~400 μm apart, minimizing crosstalk. Source and drain electrodes are patterned via maskless UV lithography, with a 20 nm Al_2O_3 insulating layer deposited by atomic layer deposition (ALD) at electrode intersections to suppress leakage currents. The prepared MoS_2/BP/MoS_2 single device is shown in Fig. S12b. A perovskite layer is then spin-coated onto the Al_2O_3 to form a gate heterojunction dielectric, and Au top-gate electrodes are thermally evaporated through a shadow mask, completing the independently addressable 3×3 sensor array (see Fig. S12a). Each unit operates with precise control, enabled by this hierarchical fabrication strategy.

We clarify that each pixel in the sensor array integrates a bidirectional phototransistor capable of generating tunable positive or negative photoconductivity states in response to visible (532 nm) or near-infrared (1064 nm) light stimuli. To demonstrate this capability, we projected laser light (532 nm and 1064 nm) through an "H"-shaped mask onto the array. As shown in Fig. S12c,d, the photoresponse currents of individual pixels were mapped spatially, revealing distinct "H" patterns for each wavelength. The 532 nm illumination induced a positive photoconductivity state, while the 1064 nm excitation triggered a negative photoconductivity transition, consistent with the bandgap-engineered heterojunction design. These analog current signals were then converted to voltage outputs via a custom peripheral circuit,

ensuring compatibility with standard image-processing algorithms for real-time pattern reconstruction.

From the perspective of imaging, Fig. S12e-g respectively demonstrates the infrared imaging, visible imaging and fusion image for the example letter "H", by the proposed device and mechanism. It can be found that infrared and visible image respectively only show the partly characteristic of the letter "H" in Fig. S12e,f. By capitalizing on the proposed device’s capability to exhibit distinct responses under visible and infrared light, we developed a tailored cell-level pixel fusion mechanism. The resulting array achieves pixel-level fusion of infrared and visible images, as demonstrated in Fig. S12g, which exhibits the combined features of both modalities. This result validates the feasibility and effectiveness of the proposed device and fusion mechanism.

$$\begin{array}{cc} \text{x-direction} & \text{y-direction} \\ \begin{bmatrix} -1 & 0 & 1 \\ -2 & 0 & 2 \\ -1 & 0 & 1 \end{bmatrix} & \begin{bmatrix} -1 & -2 & -1 \\ 0 & 0 & 0 \\ 1 & 2 & 1 \end{bmatrix} \end{array}$$

Sobel Operator

Figure S13. The used edge extraction operator in the edge extraction circuits.

In the edge extraction application, sobel operator can effectively and accurately compute the gradient magnitude and direction for each pixel in the image. Moreover, it implicitly performs lightweight Gaussian smoothing on the image (with higher weights assigned to the central pixel), which reduces noise interference in gradient calculation while maintaining edge sharpness. Comparing with other operator like prewitt and roberts, sobel has better anti-noise capability while acquiring good trade-off between computing accuracy and computing cost. In the gradient computing of edge extraction, the sobel need to be calculated with image, the essential of the computing gradient is convolution operation. Therefore, the principle of the edge extraction circuits is similar with the next convolution layer circuit.

Initially, the device-based array accomplishes perception, storage, MVM operation, and image graying. The corresponding receptive field, termed as the minor device array, computes the image gradient through OA. In the proposed circuit for extracting edge information, each row of convolution results between operator and image is transferred to the X-direction/Y-direction edge Extraction Module and perform add operation by the use of OA, which has the following principle:

$$\begin{cases} V_{\mathrm{oxij}}=R\cdot\begin{pmatrix} V_X G_{i,j}+V_Y G_{i+1,j}+V_Z G_{i+2,j}-V_X G_{i,j+2} \\ -V_Y G_{i+1,j+2}-V_Z G_{i+2,j+2} \end{pmatrix} \\ V_{\mathrm{oyij}}=R\cdot\begin{pmatrix} V_X G_{i,j}+V_Y G_{i,j+1}+V_Z G_{i,j+2}-V_X G_{i+2,j} \\ -V_Y G_{i+2,j+1}-V_Z G_{i+2,j+2} \end{pmatrix} \end{cases} \#(8)$$

where V_{oxij} and V_{oyij} are the X-direction/Y-direction edge gradient volage, G_{ij} represents the equivalent conductance of the image pixel (three device connect in parallel as the mentioned before), V_X, V_Y, V_Z are value in the used operator and V_Y is twice as V_X, V_Z (Fig. S13), and they will correspond to x_i and y_i respectively in Fig. 3a. When the voltage V_X, V_Y, V_Z apply into the proposed device array, the obtained

current is the multiplication (convolution operation) result according to Kirchhoff's and Ohm's law. The obtained current is transferred into OA to perform add or substrate operation and current transform voltage operation, the final obtained voltage V_{ox} and V_{oy} are the calculated gradient. When the circuit perform edge extracting task, x-direction and y-direction gradient calculation do not perform simultaneously, the circuit is set to firstly perform x-direction gradient calculation and calculate y-direction gradient. However, in the terms of some direction calculation like x-direction, the corresponding computing process is the whole parallel. It is different from the traditional computing architecture, which its calculating gradient for each pixel is sequential computing, and it can achieve reduction in the terms of energy and latency, which is caused by the parallel computing and no-outside-signal-conversion of analog circuit computing.

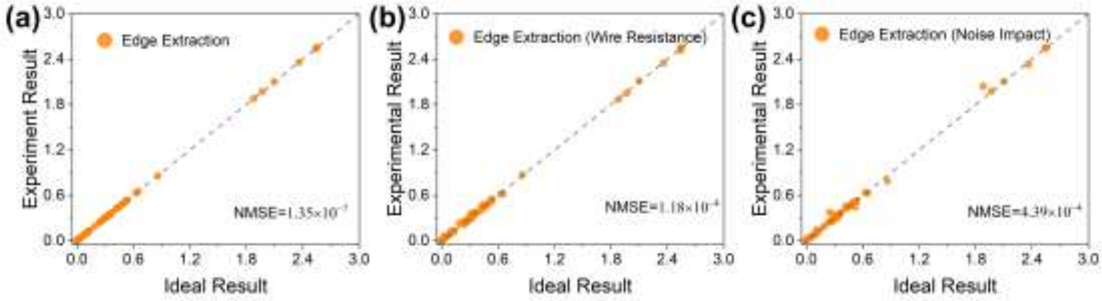

Figure S14. The evaluation results of the edge extraction circuit under different non-ideal factors impact. (a) The normal condition. (b) The 2Ω wire resistance impact. (c) The 10% input noise impact.

The above results all demonstrates the circuits has high computing accuracy and the corresponding NMSE shows each computing result has minor error with the ideal result (Fig. S14a-c).

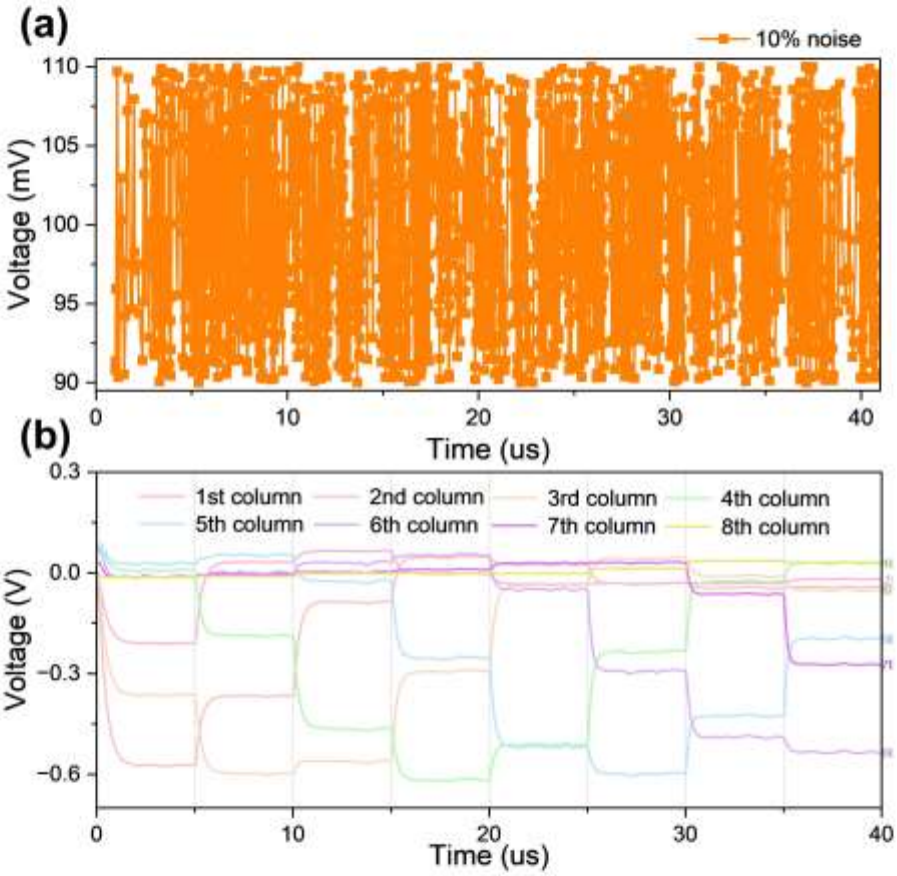

Figure S15. The evaluation results of the edge extraction circuit under noise impact. (a) The applied noise signal. (b) The circuits computing results for 5 × 5 pixels matrix example.

Figure S15a shows applied noise signal of the edge extraction circuit. Fig. S15b demonstrates the specific computing results of the circuits under noise impact, which shows that the circuits have good capacity against noise impact.

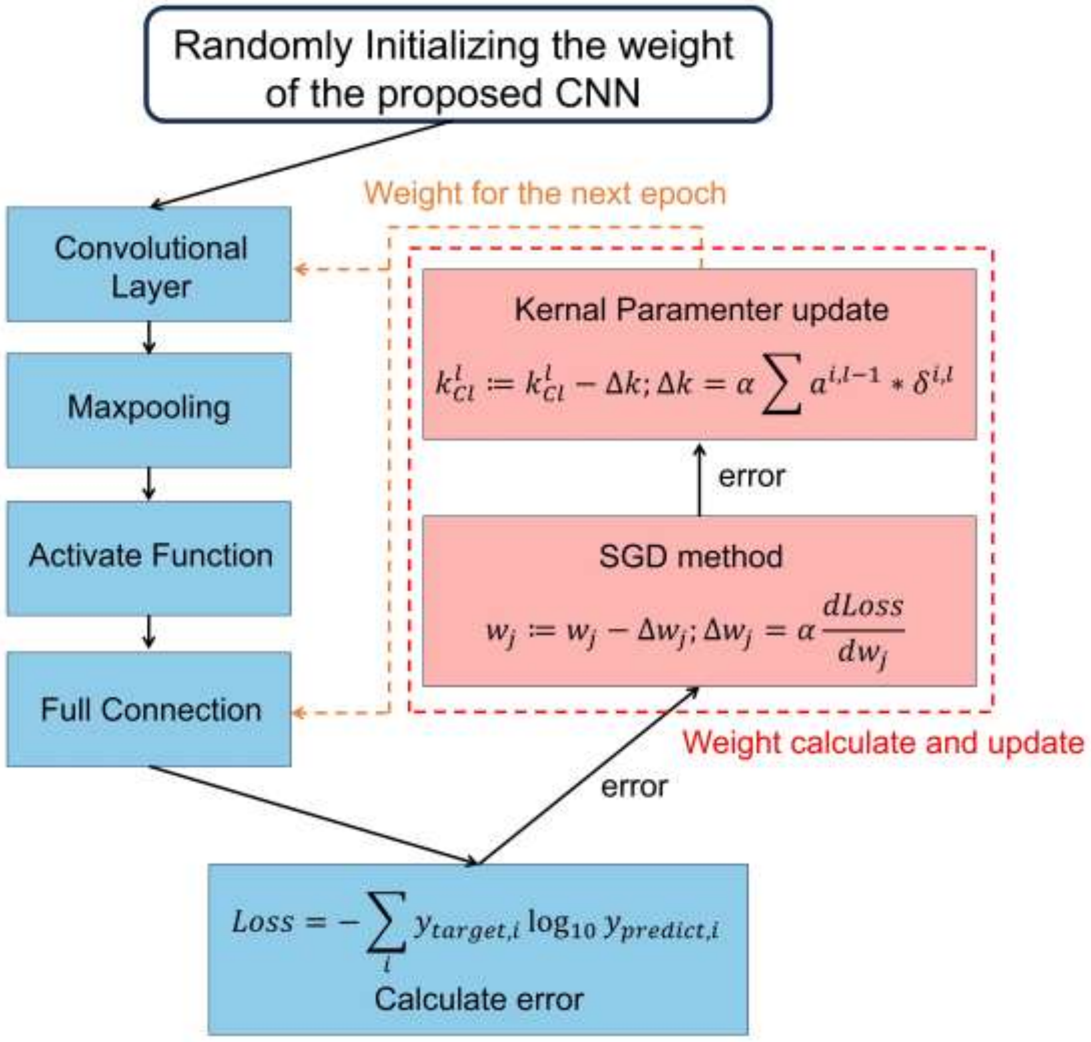

Figure S16. The training process of the utilized CNN algorithm. The stochastic gradient descent method is adopted to achieve weight update. The calculation of loss adopts cross-entropy loss function.

The training process of the employed CNN algorithm is illustrated in Fig. S16. Firstly, the weight of CNN algorithm is randomly initialized. Inputting image data-set into the proposed CNN algorithm in batch. The data subsequently pass through convolution kernel, max pooling, ReLU activation function and full connection module. The obtained result is compared with the true label and calculating error by cross-entropy loss function. Based on the loss, using SGD method and kernel parameter update method to update parameter weight of full connection layer and convolution kernel respectively. The obtained weight covers the last weight and continue the procedure until the training is complete.

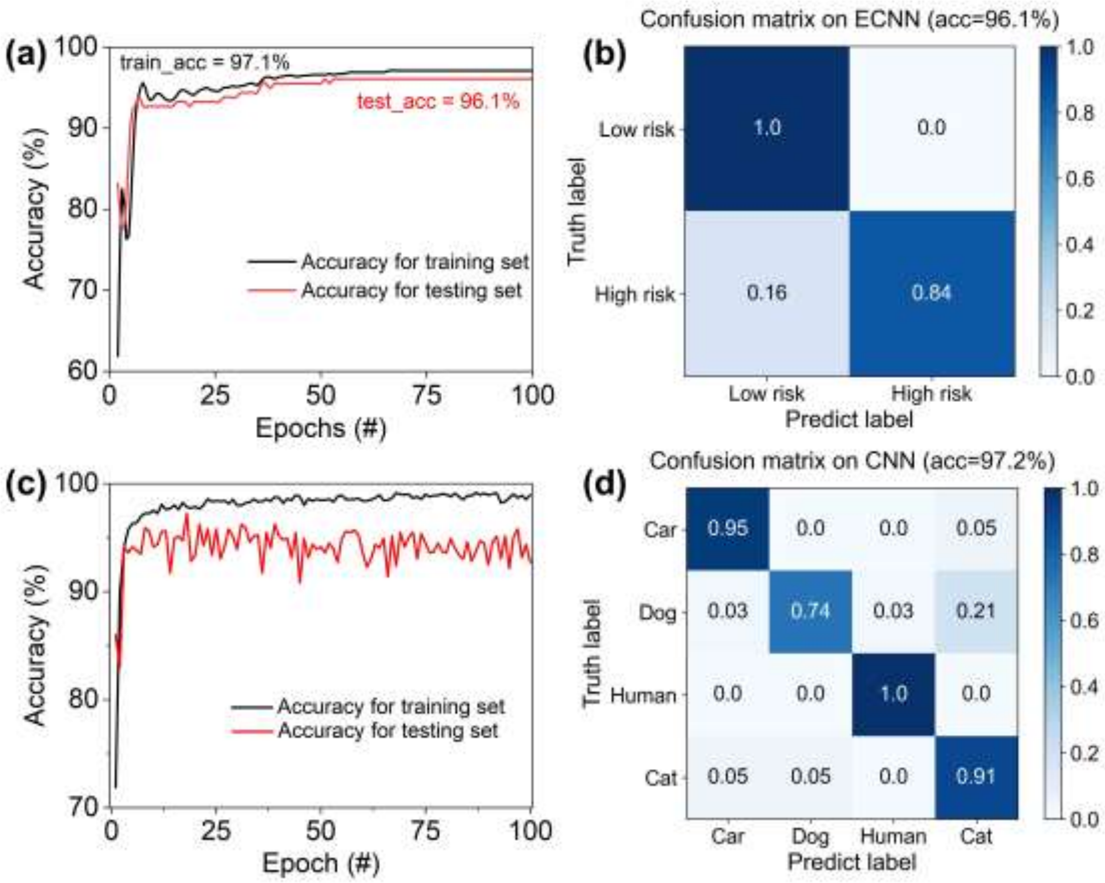

Figure S17. The test results of the proposed CNN circuits. (a) The accuracy changes for training set and test set in the different epoch during on-chip training. (b) The confusion matrix of the proposed CNN circuits for the biomimetic signal generation. (c) The accuracy changes for training set and test set in the different epoch during on-chip inference. (d) The confusion matrix of the proposed CNN circuits for the object recognition.

This work adopts the scheme that off-chip training and on-chip inference. We propose the modified CNN algorithm aiming at the requirement of the application scenario and brain-like circuits, which can be used to achieve object recognition and preparation of the biomimetic signal generation (the judgement of dangerous scenarios). The CNN trained in Fig. S17a is used for judging whether the scene is dangerous or not and assisting to generate biomimetic signal. The CNN trained in Fig. S17c is used for detecting which category of objects seen belong to in cats, dogs, humans, and cars. The over 90% testing accuracy in the above figures demonstrate that the trained network can be used to perform inference process. Specifically, the

accuracy change for training set and test in the different epoch is shown in Fig. S17a,c. Figure S17a shows that the proposed CNN has 97.1% in the terms of training set and 96.1% in the terms of test set; Figure S17c shows that the proposed CNN has 98.0% in the terms of training set and 94.1% in the terms of test set. The confusion matrix of the proposed CNN circuits for the biomimetic signal generation and object recognition is shown in Fig. S17b,d. Figure S17b shows that the proposed CNN circuits can achieve all true predictions in the terms of Low risk and 84% true accuracy in the terms of High risk; Figure S17d shows that the proposed CNN circuits respectively have 95%, 74%, 100%, 91% accuracy in terms of car, dog, human, cat recognition.

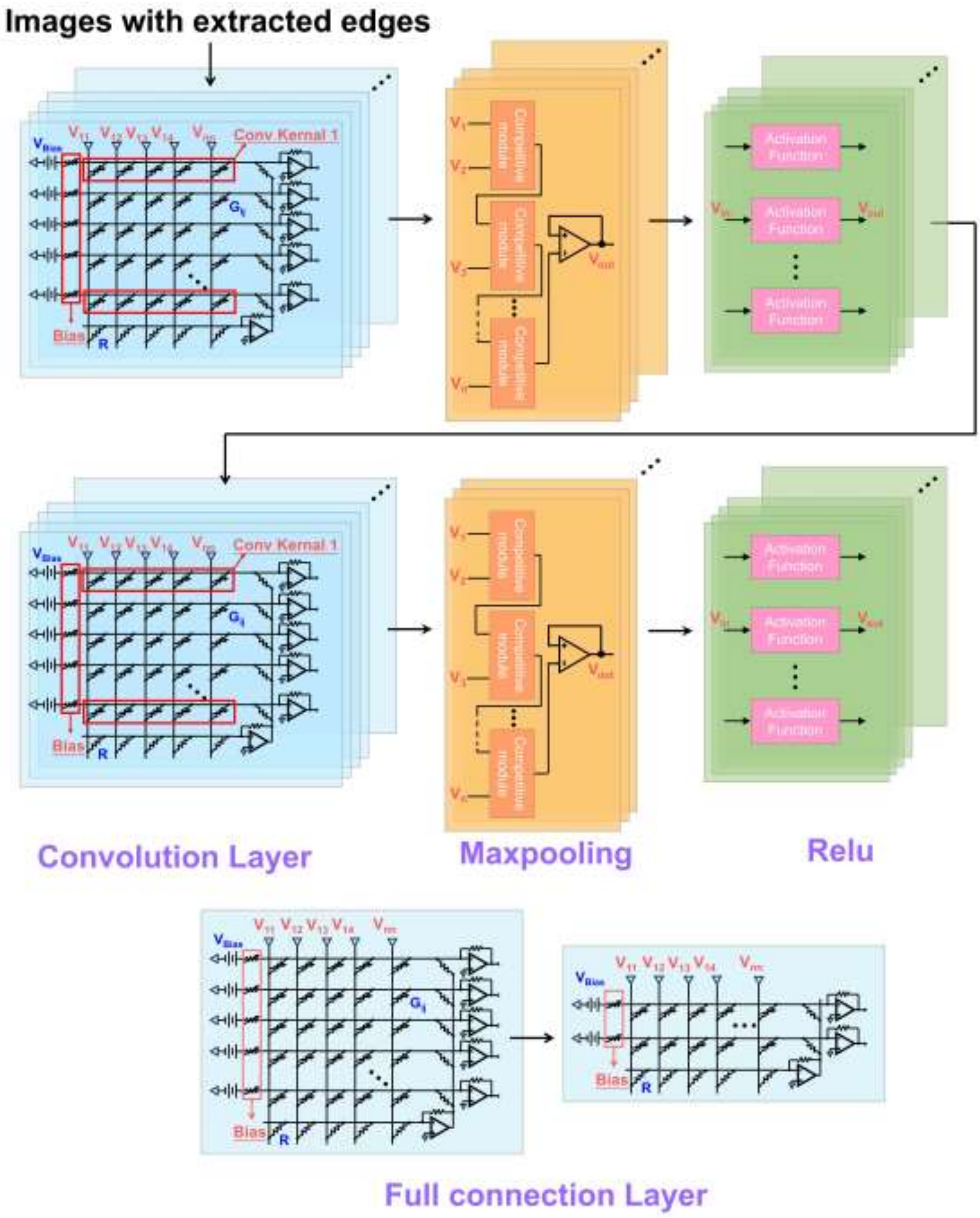

Figure S18. The overview of the proposed CNN circuits.

The CNN circuits compose of convolution layer module, maxpooling module, ReLU module and full connection layer module (Fig. S18). The convolution layer module, maxpooling module, ReLU module are composed of multiple single corresponding circuit. Moreover, the convolution layer and full connection layer module design use the proposed device, which has good storage capacity and it can map with the trained convolution kernel.

Firstly, the image needs to be processed input in the form of voltage into the

convolution layer circuit and multiple single convolution circuit operate in parallel. This is caused that the employed CNN algorithm comprises multiple convolution kernels which extracting image feature in parallel. The computing process is based on the Kichhoff's and Ohm's law, the principle of the single convolution circuit is as following:

$$V_{(i,j)}=\max\left(\left(\sum_{m=0}^{s} G_{ck}\times vec(V_{ib})\right)\cdot R\right)\#(9)$$

where $V_{(i,j)}$ represents the pixels of i-th and j-th in the processed image after k-th convolution kernel parallel process; G_{ck} represents the matrix conductance from the k-th convolution kernel weight, it has the relationship G_{ck} = [$vec(W_{conv1})$, $vec(W_{conv2})$, ... ,$vec(W_{convn})$] which $vec()$ represents vectorization and W_{convi} is the i-th convolution kernel's weight; V_{ib} is the corresponding image pixel with the convolution kernel; R is the resistance in the Trans-impedance Amplifiers (TIA), which converts current into voltage and is convenient for subsequent computations. The *max*() map with max pooling operation in CNN algorithm and it is achieved by the designed circuit in Fig. S18. The maxpooling module is designed based on the proposed competitive circuits in Fig. S18, when *n* modules operate in parallel, they can perform the whole max pooling operation. As shown in Fig. 4a, example original image and the corresponding processed image extracted edge are respectively transferred into the convolution kernel module. Following feature extraction in the convolution circuits, the voltage of the convolutional image is relayed to the fully connection layer module. The full connection layer module is similar with convolution layer circuit module, it consists of two components: the MVM execution circuits and the ReLU circuits, which correspond to the fully connected layer and the ReLU activation function, respectively. The detailed structure and principle of the circuit is depicted in Fig. S18. All weights of the utilized CNN are mapped onto the proposed device, thanks to its excellent storage capacity and stability. The full connection layer module has the following principle:

$$\begin{cases} V_{o1}=\mathrm{ReLU}[(G_{f1}V_{in})\cdot R] \\ V_{o2}=(G_{f2}V_{o1})\cdot R \end{cases} \#(10)$$

where V_{in} is the input signal which represents the convolutionalized image, G_{f1} and G_{f2} are the trained weight in the full connection layer of CNN algorithm, ReLU is the corresponding activation function. In there, the proposed circuit execute MVM operation based on the Kichhoff's and Ohm's law, the final output voltage V_{o2} is the inference result of the CNN circuit.

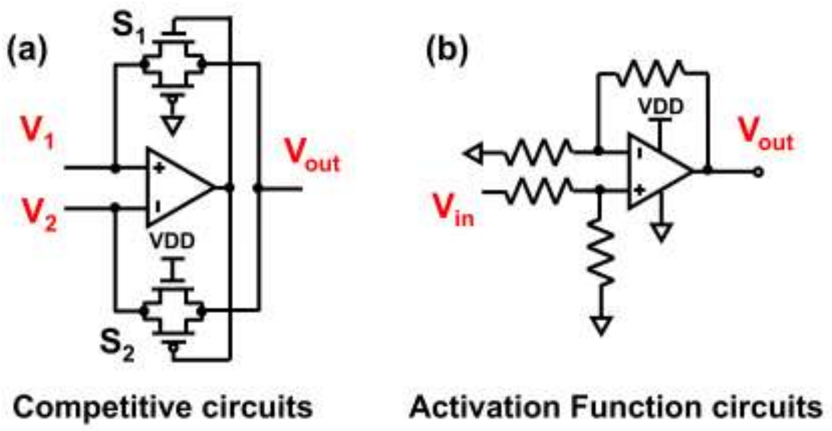

Figure S19. The structure of the used Competitive circuits and Activation Function circuits in the proposed CNN circuits. (a) The competitive circuits are used in max polling operation. (b) The activation function circuits are used to act as ReLU function module.

As illustrated in Fig. S19, the competitive circuits module composes of OA and CMOS switches, and the module has the following relation:

$$S_1=\begin{cases}1, & V_1\geq V_2\\ 0, & V_1<V_2\end{cases}, S_2=\begin{cases}0, & V_1\geq V_2\\ 1, & V_1<V_2\end{cases}, \#(11)$$

$$V_{out}=V_1\cdot S_1+V_2\cdot S_2 \#(12)$$

where the OA is used for comparing the input voltage V_1 and V_2, when V_1 is larger than V_2, the output of OA is high voltage, therefore the CMOS switch S_1 is open and S_2 is closed and the Vout is V_1, otherwise Vout is V_2. When n module operates in parallel, the modules output is the result after max pooling. The ReLU circuit module compose OA and some resistors. The circuit has as the following principle:

$$V_{out}=\begin{cases}V_{in}, & 0\leq V_{in}\leq V_{VDD}\\ 0, & V_{in}<0\end{cases} \#(13)$$

The circuits are originated from adding circuits based on the OA, the difference is that the supply voltage is set VDD and 0, which restricts the output of OA to be between VDD and 0.

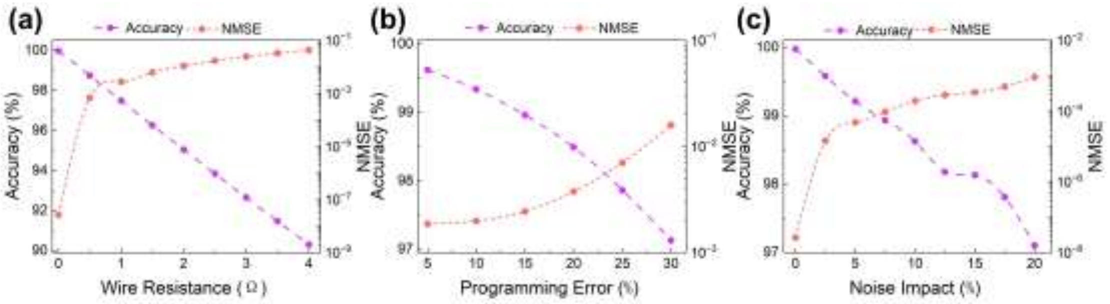

Figure S20. The evaluation results of the proposed CNN circuits under different non-ideal factors impact. (a) The different wire resistance impact. (b) The different programming error impact. (c) The different input noise impact.

The above results all demonstrates the proposed CNN circuits has good robustness against non-ideal factors impact and the corresponding NMSE also shows each computing result has minor error with the ideal result (Fig. S20).

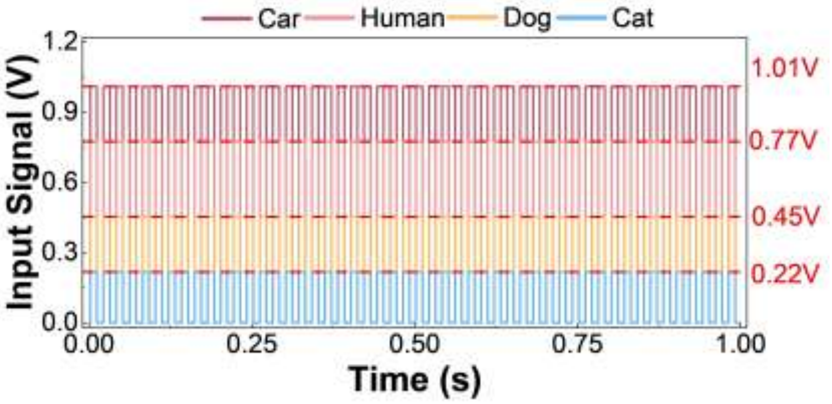

Figure S21. The input signal of the different object. The illustration of the different object's input signal to brain-like circuits, which is produced by the CNN circuits. The corresponding image is transferred into the CNN circuits, and the output is transferred into the signal conversion circuit. The output signal is the corresponding biomimetic signal.

In the brain-like circuits depicted in Fig. 4b, the threat index, time, and weather information are conveyed to the biomimetic signal converter, which subsequently generates corresponding biomimetic signals. These signals are then fed into the specially designed circuit, capable of selecting the signal with the greatest impact from among them. These operations and circuits are modeled after the thalamus. Subsequently, the selected maximum signal is compared to the V_{th}. If the maximum signal exceeds V_{th}, it indicates that external factors have reached a dangerous threshold at that moment, prompting the opening of the corresponding CMOS switch. In this case, I_{danger} represents the weighted sum of the aforementioned signals; otherwise, I_{danger} is zero, signifying safety at that instant. This process, inspired by the Amygdala, originates from the subconscious emotional changes that humans undergo in response to unexpected situations. Consequently, the IST index reflects the immediate threat situation in a driver assistance system scenario. For instance, when a pedestrian suddenly appears in front of a vehicle during driving, the image processing circuits, which are based on device arrays, CNN circuits, and brain-like circuits, can swiftly generate corresponding threat indices for decision-making, obstacle avoidance, and other operations to facilitate assisted driving. The CST index, on the other hand, reflects the threat index over a specified period. Input signals are accumulated and

integrated from time 0 to t_1, demonstrating signal accumulation. Between t_1 and t_2, C_{danger} increases as time progresses. After t_2, C_{danger} remains unchanged. This change process in C_{danger} is derived from the mechanism of the sensory cortex, which reflects the human emotional slow change during a period. Considering a driving environment scenario, adverse weather, nighttime conditions, or occasional obstacles can elevate the CST index, alerting the system to remain vigilant. Even if the IST index does not trigger an alarm at that moment, the intelligent driving system should still be prepared for such conditions (Fig. S21).

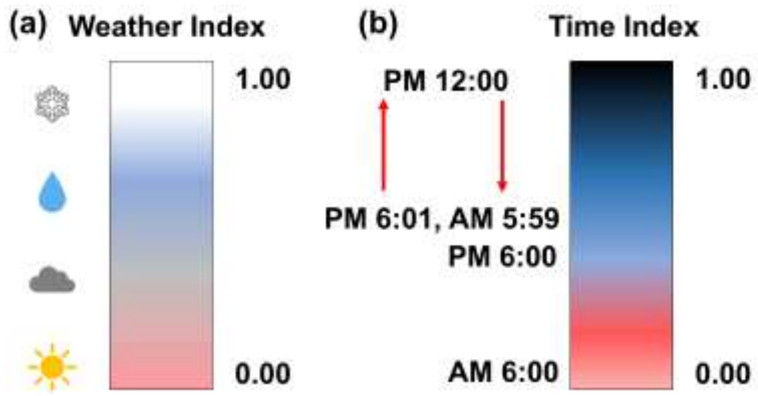

Figure S22. The quantitative and evaluation indicators in brain-like circuits. The illustration of quantitative and evaluation indicators for (a) weather and (b) time inputs in brain-like circuits.

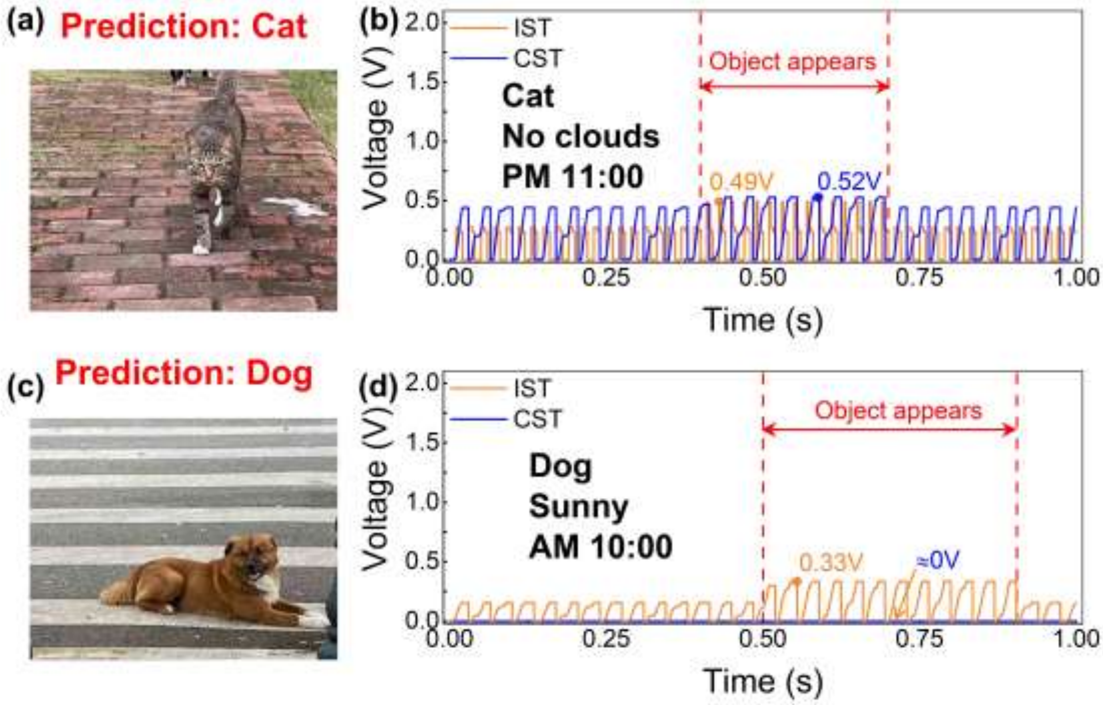

Figure S23. The results of the object recognition and decision. (a) The results of the object recognition by the proposed CNN circuits in scenario cat sudden appearing. (b) The results of the decision by the proposed brain-like circuits for cat. (c) The results of the object recognition by the proposed CNN circuits in scenario dog sudden appearing. (d) The results of the decision by the proposed brain-like circuits for dog.

When a dog appears, the IST increases but remains below the threshold, while the CST remains unchanged. This indicates that the vehicle will take precautionary measures to avoid the dog but will not trigger an alarm, and the extent of the action taken will be less pronounced compared to the previous scenarios. In the case of a sudden cat appearance, both the IST and CST elevate, with the CST notably exceeding the threshold in Fig. S23b. This is attributed to the similarity between cats and dogs, coupled with the time factor influencing driving safety. The proposed CNN circuits still have correct inference for objects classification, as shown in Fig. S23a,c.

Table S1 | Comparison of previously reported vision sensor for advanced vision system.

Device structure	Wavelength [nm]	Image recognition	Decision making	Full flow	Reference
Ga_2O_3:Sn	254	Yes	No	No	[6]
Pd/MoOx/ITO	365	Yes	No	No	[7]
BP	280, 365	Yes	No	No	[8]
In-Ga-Zn-O/Al_2O_3	405	Yes	No	No	[9]
MoS_2	405-940	Yes	No	No	[10]
Pyr-GDY/Gr/PbS-QD	430, 980	Yes	No	No	[11]
Zr-$CsPbI_3$	405-650	Yes	No	No	[12]
MoS_2/BP/MoS_2/Al_2O_3/ 2D PVK	375-1064	Yes	Yes	Yes	This work

References

1. Zhang J, Li X, Xiao P *et al.* Design of optoelectronic in-sensor computing circuit based on memristive crossbar array for in situ edge extraction. *IEEE Trans Circuits Syst I Reg Papers* 2024; **71**, 3228–41.

2. Xiao P, Hong Q, Du S *et al.* Design and application of a programmable matrix determinant-solving circuit based on memristors. *Scientia Sinica Inf* 2023; **53**, 1008.

3. Siebentritt S, Igalson M, Persson C *et al.* The electronic structure of chalcopyrites—bands, point defects and grain boundaries. *Prog Photovolt: Res Appl* 2010; **18**, 390–410.

4. Ashari-Astani N, Meloni S, Salavati AH *et al.* Computational characterization of the dependence of halide perovskite effective masses on chemical composition and structure. *J Phys Chem C* 2017; **121**, 23886–95.

5. Moon HS, Hsiao K-C, Wu M-C *et al.* Spatial separation of cocatalysts on z-scheme organic/inorganic heterostructure hollow spheres for enhanced

photocatalytic H_2 evolution and in-depth analysis of the charge-transfer mechanism. *Adv Mater* 2023; **35**, 2200172.

6. Li P, Shan X, Lin Y *et al.* Tin doping induced high-performance solution-processed Ga_2O_3 photosensor toward neuromorphic visual system. *Adv Funct Mater* 2023; **33**, 2303584.

7. Zhou F, Zhou Z, Chen J *et al.* Optoelectronic resistive random access memory for neuromorphic vision sensors. *Nat Nanotechnol* 2019; **14**, 776–82.

8. Ahmed T, Tahir M, Low MX *et al.* Fully light-controlled memory and neuromorphic computation in layered black phosphorus. *Adv Mater* 2021; **33**, 2004207.

9. Qiu W, Huang Y, Kong L-A *et al.* Optoelectronic In-Ga-Zn-O memtransistors for artificial vision system. *Adv Funct Mater* 2020; **30**, 2002325.

10. Pang X, Wang Y, Zhu Y *et al.* Non-volatile rippled-assisted optoelectronic array for all-day motion detection and recognition. *Nat Commun* 2024; **15**, 1613.

11. Hou Y-X, Li Y, Zhang Z-C *et al.* Large-scale and flexible optical synapses for neuromorphic computing and integrated visible information sensing memory processing. *ACS Nano* 2020; **15**, 1497–508.

12. Shao H, Li Y, Yang W *et al.* A reconfigurable optoelectronic synaptic transistor with stable Zr-$CsPbI_3$ nanocrystals for visuomorphic computing. *Adv Mater* 2023; **35**, 2208497.
